# Site-Specific Labelling of Multidomain Proteins by Amber Codon Suppression

**DOI:** 10.1038/s41598-018-33115-5

**Published:** 2018-10-05

**Authors:** Christina S. Heil, Alexander Rittner, Bjarne Goebel, Daniel Beyer, Martin Grininger

**Affiliations:** Institute of Organic Chemistry and Chemical Biology, Buchmann Institute for Molecular Life Sciences, Cluster of Excellence for Macromolecular Complexes, Goethe University Frankfurt, Max-von-Laue-Str. 15, 60438 Frankfurt am Main, Germany

## Abstract

The access to information on the dynamic behaviour of large proteins is usually hindered as spectroscopic methods require the site-specific attachment of biophysical probes. A powerful emerging tool to tackle this issue is amber codon suppression. Till date, its application on large and complex multidomain proteins of MDa size has not been reported. Herein, we systematically investigate the feasibility to introduce different non-canonical amino acids into a 540 kDa homodimeric fatty acid synthase type I by genetic code expansion with subsequent fluorescent labelling. Our approach relies on a microplate-based reporter assay of low complexity using a GFP fusion protein to quickly screen for sufficient suppression conditions. Once identified, these findings were successfully utilized to upscale both the expression scale and the protein size to full-length constructs. These fluorescently labelled samples of fatty acid synthase were subjected to initial biophysical experiments, including HPLC analysis, activity assays and fluorescence spectroscopy. Successful introduction of such probes into a molecular machine such as fatty acid synthases may pave the way to understand the conformational variability, which is a primary intrinsic property required for efficient interplay of all catalytic functionalities, and to engineer them.

## Introduction

**Figure 1:**
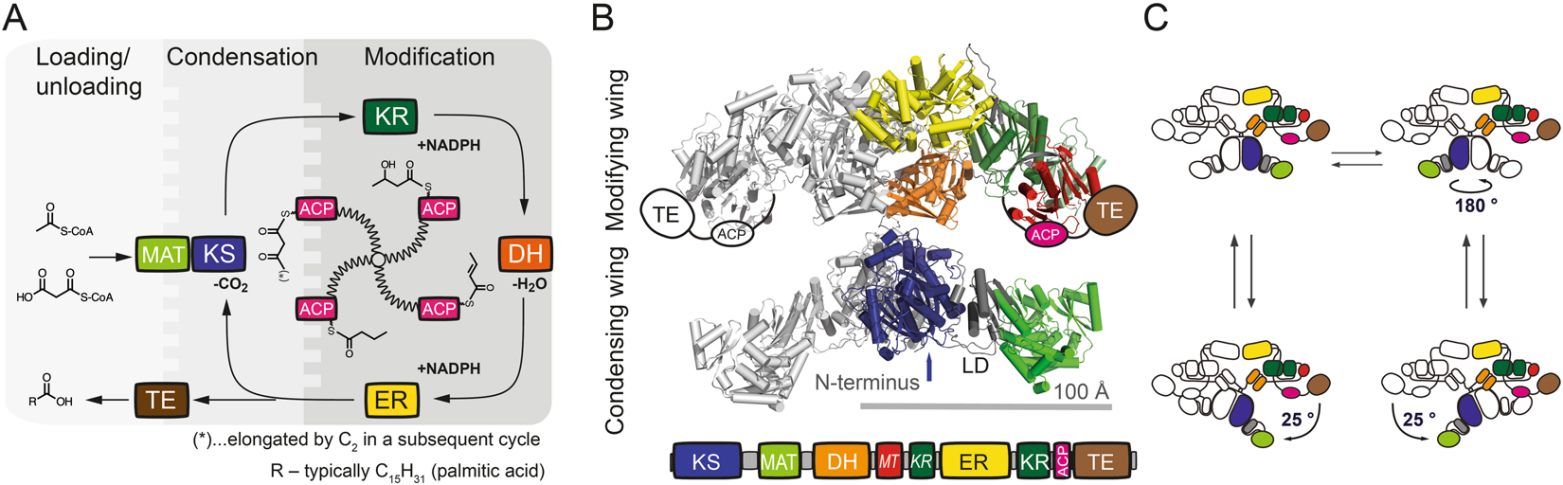
Overview of animal fatty acid synthesis. (**A**) Fatty acid synthesis as occurring in animals. The fatty acid, typically palmitic acid, is produced from the substrates acetyl-CoA, malonyl-CoA and NADPH. The acetyl moiety is sequentially elongated and modified by several domains until a certain chain length (C_16_) is reached and the final product is released from the enzyme as a free fatty acid. During the whole process, all intermediates remain covalently attached to the enzyme, mainly to the ACP domain, which requires a high conformational freedom of FAS to facilitate productive interactions between the ACP domain and all catalytically active sites. Domain nomenclature: KS (ketoacyl synthase), KR (ketoacyl reductase), DH (dehydratase), ER (enoyl reductase), ACP (acyl carrier protein), TE (thioesterase), MAT (malonyl/acetyltransferase). (**B**) Cartoon depiction of the dimeric “X”-shaped structure of porcine FAS^6^. α-Helices are shown as cylinders. One half of the dimer is coloured according to the attached domain overview. Owing to their high positional variability, ACP and TE could not be traced in electron density, but are schematically drawn for clarity. *KR* and *MT* (methyltransferase) refer to non-catalytic folds, which have structural tasks and may confine the ACP during substrate shuttling. (**C**) Conformational dynamics of animal FAS. Swinging and swivelling motions around the flexible hinge region have been observed by single particle EM and high-speed atomic force microscopy^8,15^. Full rotation of the condensing wing by 180° was further confirmed by mutagenesis studies^49^.

Fatty acid synthases type I (FASs) are large and complex multidomain enzymes that are responsible for cytosolic *de novo* fatty acid synthesis^1,2^. Evolutionarily related to FAS are polyketide synthases type I (PKSs) that synthesize polyketides, which account for one of the largest classes of natural products^3,4^. In FASs and PKSs, multiple catalytic sites collaborate in the stepwise assembly of simple building blocks to produce their final products (Fig. 1A)^5^. An important feature during biosynthesis is that substrates and intermediates remain covalently bound to the enzyme at all time. This implies that the acyl carrier protein (ACP) domain, shuttling substrates between the different catalytic domains, undergoes large positional variability, and is complemented by conformational flexibility within and across the FAS and PKS modules^6–9^.

While the overall architecture of type I PKSs has not yet been firmly elucidated^10,11^, high resolution data of FASs in different structural arrangements are available^6,12^. As observed in 3.2 Å model X-ray crystal structure on FAS from pig, animal FAS assembles into an intertwined dimer of approximately 540 kDa, adopting an “X”-shaped conformation (Fig. 1B). Although the ACP and TE domains could not be traced in the electron density, it becomes apparent from the model that a positionally variable ACP alone is not able to reach every catalytic centre. This paradox was already described by Hammes *et al*. in an early fluorescence resonance energy transfer (FRET) study on chicken liver FAS^13,14^. More detailed insights into the conformational versatility of animal FAS were finally given by a recent negative stain electron microscopy (EM) study on rat FAS, high-speed atomic force microscopy on insect FAS and by computational modelling with porcine FAS data^7,8,15^. These studies revealed large conformational changes within the enzyme with complete relative rotational and swinging freedom between the condensing and processing wing (Fig. 1C).

As many biochemists are interested in understanding these individual reactions during product synthesis, we seek to establish spectroscopic methods at the single-molecule level, to monitor a continuous spectrum of stochastic conformational motions in proteins^16,17^. An integral aspect of spectroscopic methods is the modification of proteins with labels. Conventional techniques, such as labelling naturally occurring or mutationally introduced cysteines via maleimide chemistry^18^, are not applicable for animal FAS, since the large complex features many native cysteines, including active site cysteines. Our method of choice was therefore the genetic encoding of non-canonical amino acids (ncAAs) through the amber codon suppression technology^19,20^. Such ncAAs carry orthogonal functional groups, which can be used to site-specifically attach spectroscopic labels by post-translational modification.

To the best of our knowledge, the introduction of ncAAs and the subsequent bioconjugation with a fluorophore have neither been reported for megasynthases, such as FASs and PKSs, nor for any other multidomain protein of such size. We therefore established a systematic approach, in which the screening of amber codon suppression systems, ncAA insertion sites and fluorophore click protocols can be performed with an authentic system of low complexity in an ACP-GFP fusion protein that promises a high success rate in upscaling for the production of the full-length protein. In particular, we have applied this labelling scheme to a murine FAS (mFAS) that is usually difficult to tag without compromising the biological function. The results demonstrate that ncAA labelling is a promising new tool that can be used to characterize the dynamics of large megasynthases and may help in better understanding the molecular mechanism of such biosynthetic machineries.

## Results

### Screening of the amber codon suppression toolbox

**Figure 2:**
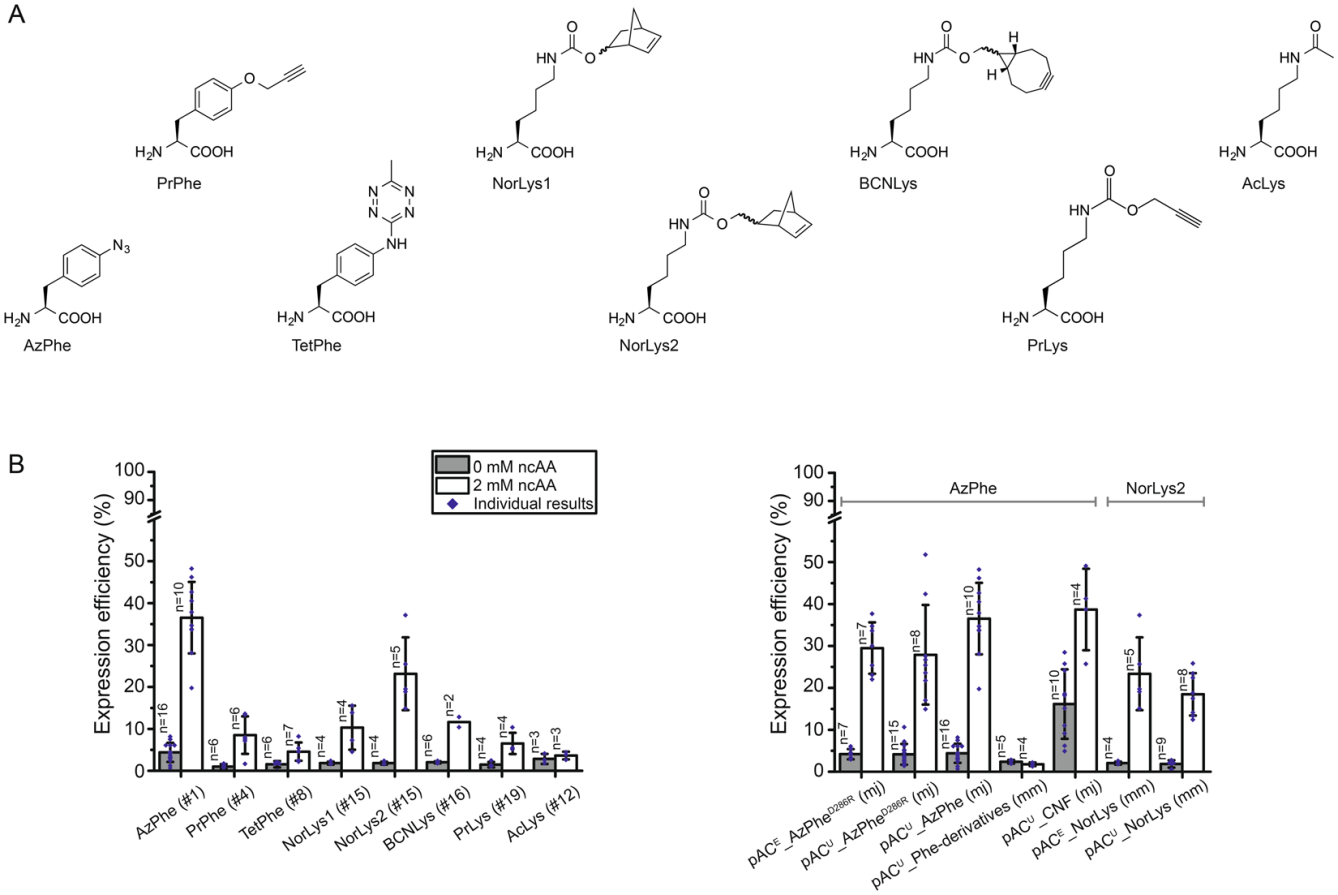
Amber codon suppression at site Leu54 in the ACP-GFP fusion construct screened in reporter assay. (**A**) Overview of ncAAs used in this study. (**B**) Best expression efficiency of different ncAAs (left panel) and comparison of some representatives of the screening (right panel). Respective plasmids used for incorporation of ncAAs are indicated by plasmid number (#; listed in Supplementary Table S2). A compilation of all results from the reporter assay can be found in Supplementary Fig. S2. Expression efficiency is read out by GFP fluorescence of 2 mL *E*. *coli* cell cultures and compared to wild type reference (taken as 100%). For incorporation, 2 mM ncAAs were supplemented to the medium. Cultures lacking ncAAs were taken as negative control to determine background signal. Dots refer to values of the biological samples. The averages of biological samples are plotted together with standard deviations. Technical errors were below 10%.

Out of a growing repertoire of ncAAs that are introduced by amber codon suppression, we limited our screening to eight different ncAAs with functional groups that can be used for bioconjugation with click chemistry or oxime formation (Fig. 2A; for syntheses of respective ncAAs, see Supplementary Methods)^19^. Azido- and propargyl-functional groups can for example be used in copper(I)-catalysed alkyne-azide cycloadditions (CuAACs)^21^. Since copper is critical for the stability of the protein, we focused on copper-free click chemistry, like the strain-promoted alkyne-azide cycloaddition (SPAAC)^22^, with azido- and bicyclononyne (BCN)-functional groups, and the inverse electron-demand Diels-Alder cycloaddition (IEDDAC)^23^, with tetrazine- and norbonene-functional groups. Additionally, we also tested incorporation of the ncAA AcLys, as the acyl-functional group can be bioconjugated in oxime formation^24^, and acetylation of lysines naturally occurs as post-translational modification in animal FAS^25^.

Further, we compared two common suppressor vectors pUltra and pEVOL (see Supplementary Fig. S1), published by Schultz and coworkers^26,27^, and several evolved aminoacyl-tRNA synthetase (aaRS)/tRNA pairs from *Methanococcus jannaschii*, *Methanosarcina mazei* and *Methanosarcina barkeri* for their performance. The cloning procedure of suppressor vectors pAC^U^ and pAC^E^ (based on original pUltra and pEVOL, respectively) is described in detail in the Supplementary Methods. Supplementary Table S1 lists all primers used for cloning, and Supplementary Table S2 summarizes all suppressor plasmids and evolved aaRS of this study.

To identify the optimal pair of suppression system and ncAA, we established a reporter assay. The screening was performed on a fusion construct of ACP from mFAS with GFP (ACP-GFP), placing the amber mutation in a disordered and non-conserved loop region at the Leu54 site (equivalent to Leu2166 in mFAS, UniProtKB accession code P19096), using the homologous rat ACP structure (PDB: 2png) as template. Incorporation efficiency was read out at 2 mL scale by recording the fluorescence of *E*. *coli* cell cultures expressing different ACP-GFP mutants. Cultures lacking the ncAA in the medium were taken as negative controls to determine the background signal (Fig. 2B). Negative samples showed a fluorescence level of up to 4% of the wild type reference, with one exception, suppressor plasmid pAC^U^_CNF showing a high fluorescence level of 16 ± 8%. High incorporation efficiencies were observed for AzPhe^28^ (37 ± 9%) and NorLys2^29,30^ (23 ± 9%). The ncAA BCNLys^31^ was incorporated with 12 ± 2% efficiency, all other ncAAs (PrPhe^32^, TetPhe^33^, NorLys1^23^ and PrLys^34^) showed efficiencies below 10%, and AcLys^35^ was hardly incorporated at all. Comparing the suppressor plasmids pAC^E^ and pAC^U^, the plasmid pAC^E^ seemed to be slightly more efficient in our set-up than its pAC^U^ counterpart (30 ± 6% pAC^E^_AzPhe^D286R^ vs. 28 ± 12% pAC^U^_AzPhe^D286R^ and 23 ± 9% pAC^E^_NorLys vs. 18 ± 5% pAC^U^_NorLys). The D286R mutation in the aminoacyl-tRNA synthetase of *M*. *jannaschii*^36^, which was reported to have a beneficial effect, did not improve incorporation efficiencies in our hands (28 ± 12% pAC^U^_AzPhe^D286R^ vs. 37 ± 9% pAC^U^_AzPhe). Comparing the two orthogonal systems, the tyrosyl-tRNA synthetase derived from *M*. *jannaschii* (mjTyrRS) seemed to be more efficient in our set-up than the pyrrolysyl-tRNA synthetases of *M*. *mazei* or *M*. *barkeri* (mmPylRS or mbPylRS, respectively) (e.g. 37 ± 9% pAC^U^_AzPhe (mj) vs. 18 ± 5% pAC^U^_NorLys (mm)). We also tested two less specific suppressor vectors, which incorporate multiple ncAAs. While the suppressor plasmid pAC^U^_Phe-derivatives of *M*. *mazei*^37^ failed to incorporate any phenylalanine derivatives, the suppressor plasmid pAC^U^_CNF from *M*. *jannaschii*^38,39^ showed high incorporation efficiencies of 39 ± 10%, but suffered from relatively high fluorescence of the negative control (16 ± 8%), indicating unspecific incorporation of endogenous amino acids. A compilation of all results from the reporter assay screening can be found in Supplementary Fig. S2.

### Screening of ncAA incorporation sites

**Figure 3:**
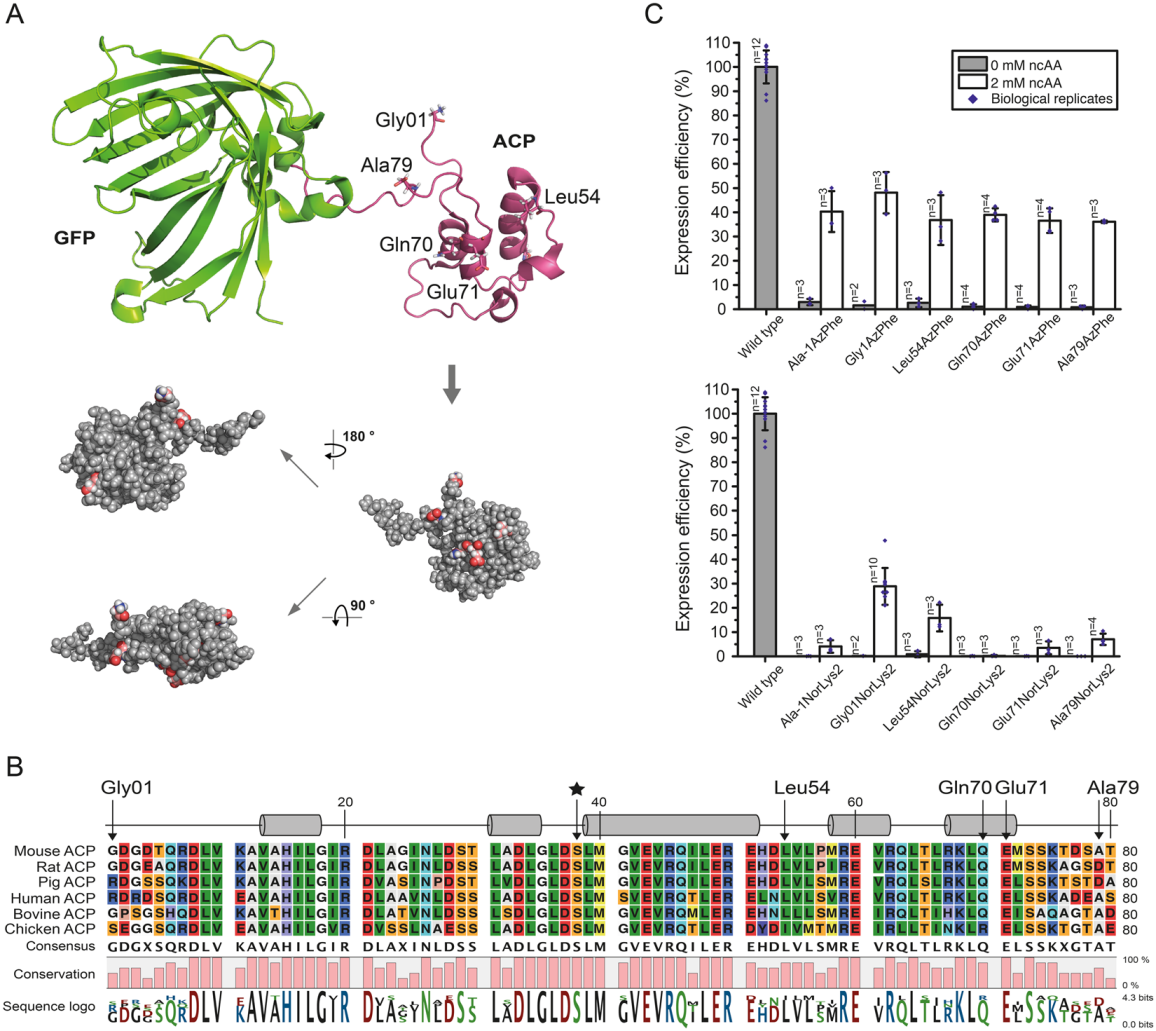
Screening of amber codon mutation sites. (**A**) Cartoon representation of the ACP-GFP fusion construct (upper panel; pink: rat ACP (PDB: 2png) and green: eGFP (PDB: 2y0g)) used in the reporter assay. The five amber mutation sites are labelled and depicted in stick representation (Gly01, Leu54, Gln70, Glu71 and Ala79). Different orientations of the ACP domain (shown in a sphere-filling model) demonstrate the positioning of all amber mutation sites on the surface of the domain (lower panel). Amber mutation sites are coloured in red. (**B**) Sequence alignment of six different ACP domains of animal FASs. UniProtKB accession codes: mouse FAS: P19096; rat FAS: P12785; pig FAS: A5YV76; human FAS: P49327; bovine FAS: Q71SP7 and chicken FAS: P12276. The five amber mutation sites are highlighted by arrows, and a star highlights the active serine residue. Secondary structure elements received from the rat ACP model (PDB: 2png) are depicted (α-helices shown as cylinders). (**C**) Expression efficiencies of six different AzPhe mutants (upper panel) and six different NorLys2 mutants (lower panel) in comparison to the wild type reference, read out by the GFP fluorescence of 2 mL cultures of *E*. *coli* cells. For incorporation, 2 mM ncAAs were supplemented to the medium. Cultures lacking ncAAs were taken as negative control to determine background signal. The averages of biological replicates are plotted together with standard deviations and the distribution of individual values is indicated as dots. Technical errors were below 10%.

As it has been reported before that the specific position of an amber mutation has major effects on incorporation efficiencies^40^, we used the most promising systems from the initial screening to compare incorporation efficiencies at different sites (pAC^U^_AzPhe with AzPhe as the optimal result and the respective vector pAC^U^_NorLys for NorLys2). Hence, we selected six positions in the ACP fold to test the acceptance of ncAA incorporation (Ala in the linker region between the N-terminal Strep-tag and ACP-GFP, Gly01 at the N-terminus of the mouse ACP sequence (equivalent to Gly2113 in mFAS), Leu54 in a disordered loop region (equivalent to Leu2166 in mFAS), Gln70 and Glu71 in helix 5 (equivalent to Gln2182 and Glu2183 in mFAS) and Ala79 in the linker between ACP and GFP (equivalent to Ala2191 in mFAS); see Fig. 3A,B). AzPhe was incorporated with good efficiencies (in average 38 ± 1%) throughout all amber mutation sites, whereas the incorporation efficiencies for NorLys2 were strongly dependent on the respective position (Fig. 3C). Best incorporation efficiency for NorLys2 was gained for the amber mutation site Gly01 at the N-terminus with 39 ± 13%. The amber mutation site Leu54 in the disordered loop region of ACP showed 16 ± 6% incorporation efficiency for NorLys2, whereas amber mutation site Gln70 in the last helix of ACP showed no incorporation at all. All other amber mutation sites showed incorporation efficiencies below 10%. Higher concentrations (4 mM and 8 mM) of ncAAs in the medium seemed to slightly increase incorporation efficiency of NorLys2 and slightly decrease efficiency of AzPhe (see Supplementary Fig. S3), but did not show a significant trend. Therefore, we proceeded with a concentration of 2 mM ncAA.

### Upscaling of protein production

**Figure 4:**
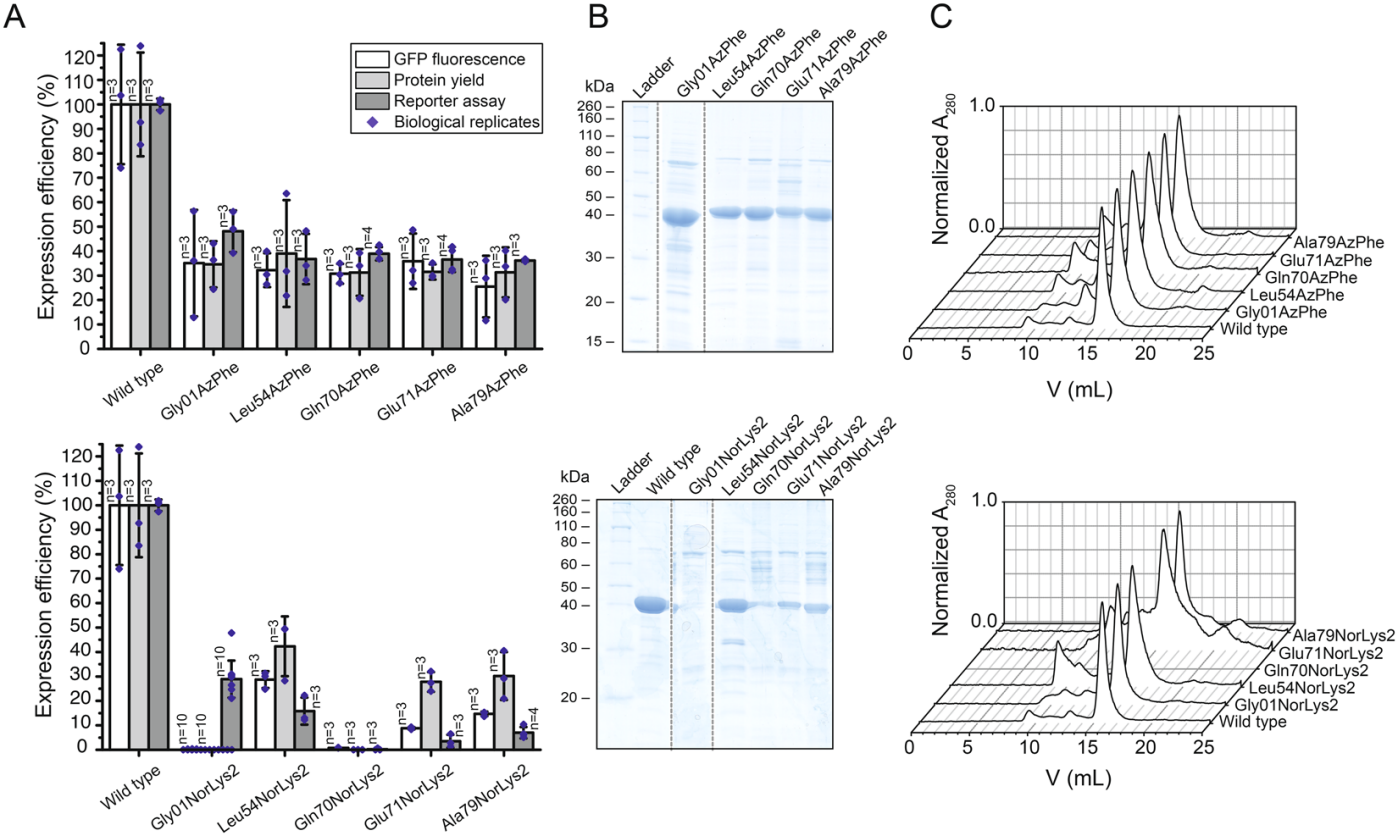
Large scale expression and purification of ACP-GFP mutants (upper panels AzPhe mutants, lower panels NorLys2 mutants). (**A**) Comparison of the results from large scale expression cultures (protein yield was read out by GFP fluorescence of a 2 mL sample and by the yield of purified protein) with previous results from the reporter assay. Data compare expression efficiency of wild type and five different AzPhe mutants (upper panel), and expression efficiency of wild type and five different NorLys2 mutants (lower panel). All expression efficiencies are related to the wild type reference. For incorporation, 2 mM ncAA were supplemented to the medium. The averages of biological replicates are plotted together with standard deviations and the distribution of individual values is indicated as dots. Technical errors were below 10%. (**B**) SDS-PAGE (NuPAGE Bis-Tris 4–12%) gel of ACP-GFP mutants purified by Ni-chelating chromatography. Lanes have been assembled for clarity (indicated by dashed lines), but scans of the original gels can be found in Supplementary Fig. S9. SDS-PAGE shows one set of purified proteins (one biological replicate). (**C**) Preparative SEC of ACP-GFP mutants performed with a Superdex 200 Increase 10/300 GL column (the set of proteins shown in (**B**)). Peaks at an elution volume of 16 mL correspond to the ACP-GFP variants. The void volume of the column is at ca. 9 mL.

In a next step, it was tested whether the selected conditions from the reporter assay could be reproduced in larger expression cultures of 200 mL. Each culture was analysed in fluorescence, as was implemented in the reporter assay, and further evaluated by the yield of purified protein. Fluorescence data was collected similarly to the reporter assay, taking a 2 mL sample of the cell culture. The ncAA AzPhe was incorporated with overall good efficiency (in average 32 ± 4%), whereas large variations were observed for incorporation of NorLys2 at different amber mutation sites (Fig. 4A). Best incorporation efficiency for NorLys2 was gained for the amber mutation site Leu54 with 29 ± 3% and no incorporation was achieved at amber mutation site Gln70. This data agreed well with the results from the reporter assay, except for the incorporation of NorLys2 in Gly01 failing at larger volume, while leading to best incorporation efficiencies in the reporter assay. We observed systematic higher values for NorLys2 and slightly lower values for AzPhe by GFP-fluorescence in the larger expression culture as compared to the reporter assay.

**Figure 5:**
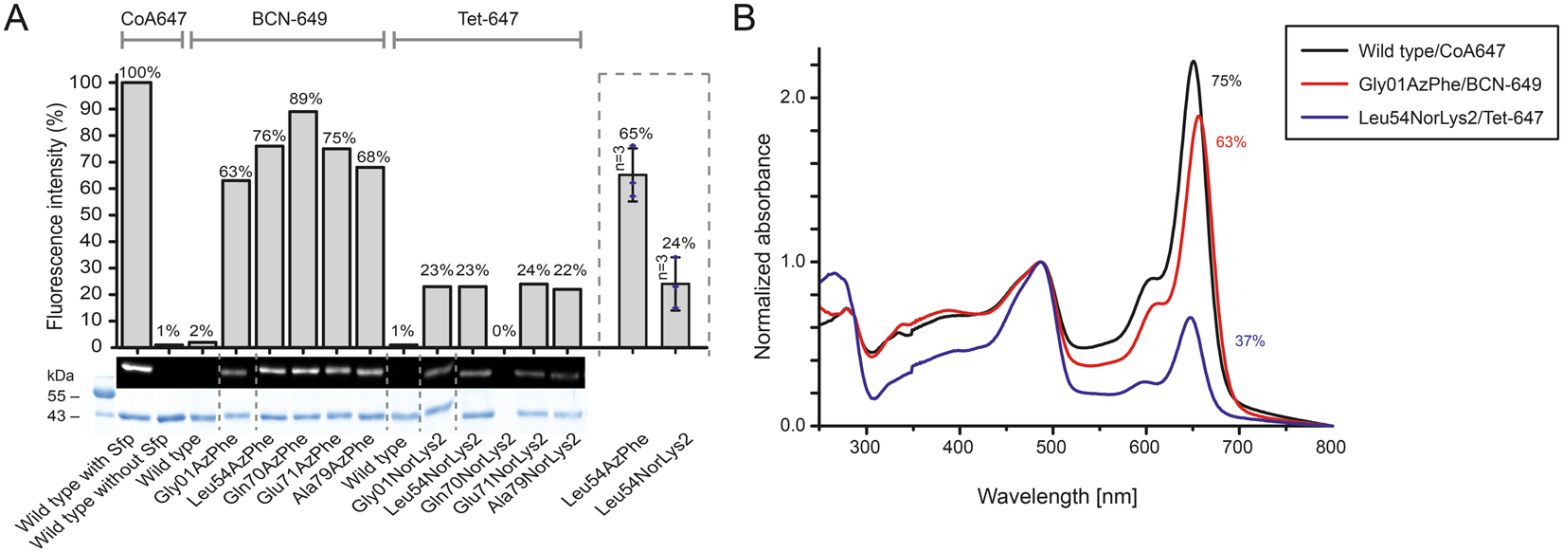
Fluorescent labelling of ACP-GFP mutants. (**A**) DOL of ACP-GFP mutants in respect to the amber mutation site determined by relative in-gel fluorescence intensities at wavelength 650 nm. The ACP-GFP construct was enzymatically modified by a fluorescent CoA647-label with Sfp and served as the wild type reference. Hence, it was put to 100% fluorescence intensity. AzPhe mutants were labelled with 80 equiv. of BCN-POE_3_-NH-DY649P1 (BCN-649), NorLys2 mutants were labelled with 80 equiv. of 6-methyl-tetrazine-ATTO-647N (Tet-647) in 10 µL reaction volume. All fluorescence intensities were corrected by the quantum efficiency of the respective fluorophore and correlated to the protein bands of the Coomassie-stained gel (lanes have been assembled for clarity, as indicated by dashed lines). Scans of the original gels are presented in Supplementary Fig. S10. Biological replicates were performed for the Leu54 mutants, comparing DOL determined for three parallel labelling reactions analysed on different fluorescent gels (inserted box). Individual results were gathered from gels in A and Supplementary Fig. S4. (**B**) DOL determined by UV-Vis spectroscopy. 25 equiv. of fluorophore were used in labelling reactions of ACP-GFP mutants in 50 µL reaction volume. Free fluorophore was removed by purification over Ni-NTA magnetic beads. UV-Vis absorbance spectra were normalized to GFP absorbance at wavelength 485 nm. DOL is read out by comparing absorbance of the fluorophore at 650 nm to absorbance of GFP at 485 nm.

For comparing protein yields, cells received from 200 mL expression cultures were lysed and proteins were isolated by Ni-chelating chromatography. Compared to ACP-GFP at 53 ± 15 mg, the positive mutants were expressed in average with 14 ± 3 mg yield, which corresponds to about 25% of the wild type protein yield (Fig. 4B). The incorporation efficiency quantified by protein yield correlated well with the trends of the fluorescence data. AzPhe was again incorporated with overall good efficiency (in average 33 ± 4%), whereas NorLys2 performed differently throughout the amber mutation sites (Fig. 4A). The optimal site for NorLys2, Leu54 in the disordered loop region, showed 42 ± 12% incorporation efficiency, and even the amber mutation sites Glu71 and Ala79 gave up to 30% incorporation efficiency. Again, no incorporation of NorLys2 at the amber mutation site Gln70 was monitored. We note that NorLys2 mutants led to higher protein yields than expected from fluorescence intensities of cell cultures, which cannot be explained with the collected data. We note that protein yield determined after Ni-chelating chromatography is only an estimate, as some impurities can still be seen in the gels (Fig. 4B) and size exclusion chromatography (SEC) (Fig. 4C). Also the loss of protein during purification may alter the amount of purified protein. The quality of proteins was analysed by SEC and mass spectrometry (MS) (see Supplementary Data). The elution profiles of the different ACP-GFP mutants matched very well with the wild type SEC spectrum (Fig. 4C). In MS analysis, a mass shift for the Gln70AzPhe mutant was observed, although we were able to specifically label this mutant in further experiments (see Fig. 5), which confirms presence of the azido group at this stage. Instability of the azido group during protein analysis may account for the reduced molecular weight, but cannot explain why this mass shift is only observed for this mutant. Owing to this inconsistency, the Gln70 position was considered unreliable and not chosen in full-length protein constructs.

### Fluorescent labelling of ACP-GFP

In first experiments, we screened click kinetics for our target protein ACP-GFP (see Supplementary Fig. S4) and received a suited condition of 2 h of incubation at room temperature with 80 equiv. of fluorophore in 10 µL reaction volume for both the SPAAC (AzPhe mutant BCN-POE_3_-NH-DY649P1 conjugate) and the IEDDAC (NorLys2 mutant 6-methyl-tetrazine-ATTO-647N conjugate). For determining the degree of labelling (DOL) by in-gel fluorescence, ACP-GFP enzymatically modified with a fluorescent CoA-label by a 4′-phosphopantetheinyl transferase (Sfp)^41^ was used as reference. For better comparison, the different fluorescence intensities were corrected by the respective quantum efficiencies. Three different fluorophores were used in this experiment: DY647P1 at the CoA-label (quantum efficiency 30% according to the manufacturer), DY649P1 at the BCN-label (quantum efficiency 30% according to the manufacturer) and ATTO 647 N at the tetrazine-label (quantum efficiency 65% according to the manufacturer). The sample of ACP-GFP enzymatically modified by a fluorescent CoA-label showed highest fluorescence and was assumed to be quantitatively labelled, based on previous reports^42^, and thus set to 100% as the wild type reference. As this aspect has a great impact on our data, we performed additional experiments to verify that no unmodified protein is present in the reference sample (see Supplementary Fig. S5). All fluorescence intensities were further correlated to the intensity of the protein bands of the Coomassie-stained gel. In average, the AzPhe mutants clicked more efficiently than the NorLys2 mutants (74% over 23%) (Fig. 5A). As in-gel fluorescence is always determined relative to the wild type reference, accurate comparison of intensities between different gels is difficult, and thus we observed variations in the DOL. Biological replicates of the labelling reaction for both Leu54 mutants were performed and compared (see inserted box in Fig. 5A). The DOL of the Leu54AzPhe mutant was in average 65 ± 10% and of the Leu54NorLys2 mutant 24 ± 10% (see Fig. 5A). With respect to the given error margin of approximately 20%, no statement to a position dependency of the labelling reaction can be made.

The DOL was alternatively determined by spectroscopy with samples Gly01AzPhe and Leu54NorLys2, after removal of excess free fluorophore by purification over Ni-NTA magnetic beads (see Supplementary Fig. S6). In a single experiment, these proteins were clicked with 25 equiv. of fluorophore in 50 µL reaction volume and the labelling efficiency was monitored with UV-Vis absorbance spectra. For the wild type reference, a DOL of 75% was determined, whereas the Gly01AzPhe mutant showed 63% DOL and the Leu54NorLys2 mutant showed only 37% DOL (Fig. 5B). We explain the difference in DOL determined by in-gel fluorescence intensities and UV-Vis absorbance spectra as originating from different reaction conditions and sample preparations performed for analysis in SDS-PAGE and spectroscopy (see Fig. 5A,B). The quantum efficiency is determined for the free fluorophore and may be differently affected by the microenvironment within the native and the denatured protein. Data may also indicate photobleaching of the CoA 647 conjugate in the reference sample (see Supplementary Fig. S5).

### Labelling of and initial experiments on full-length mFAS

**Figure 6:**
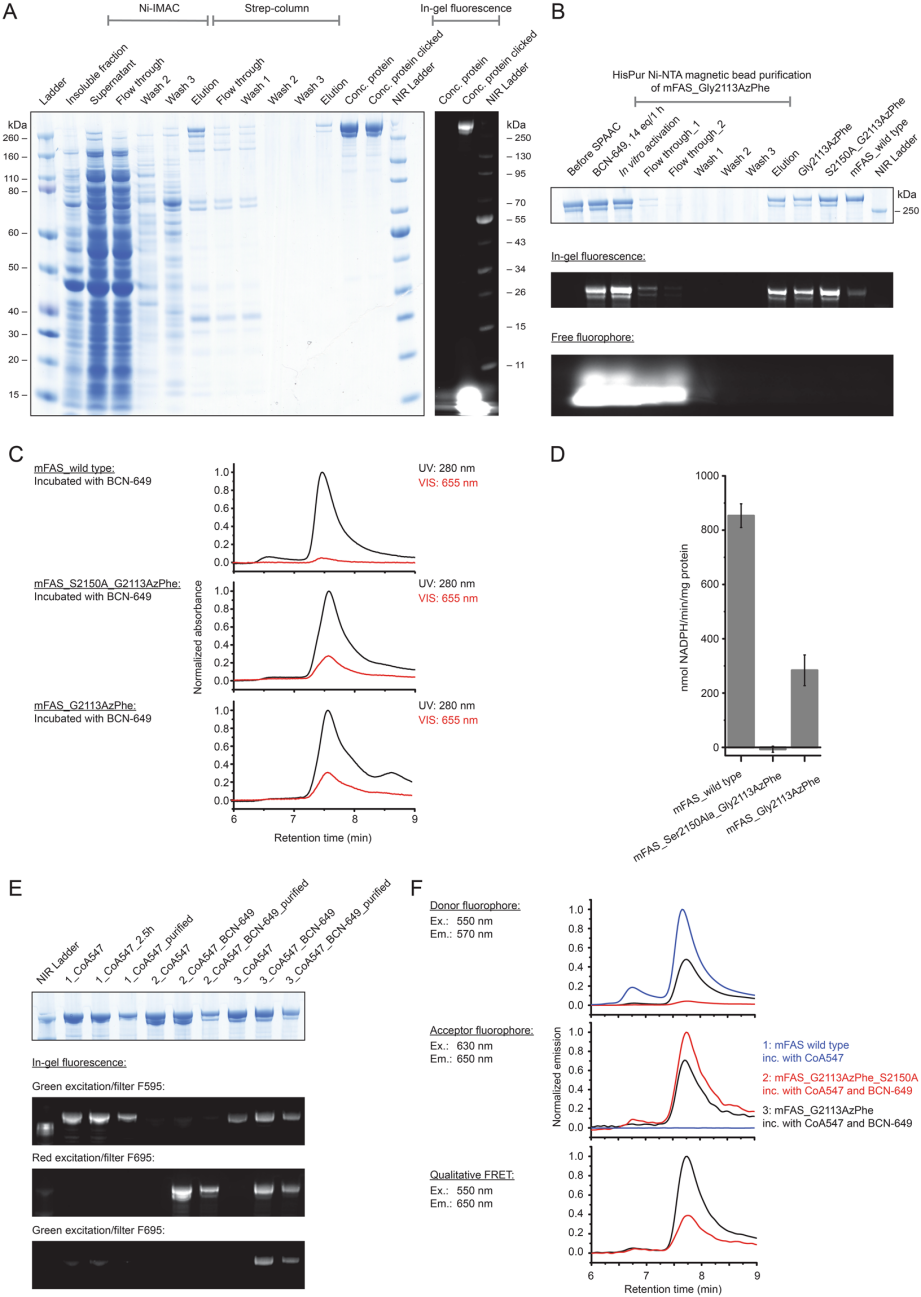
Physicochemical analysis of fluorescently labelled mFAS. (**A**) SDS-PAGE (NuPAGE Bis-Tris 4–12%) of a representative purification of the ncAA-modified mFAS mutant (Gly2113AzPhe with additional ACP knock-out mutation Ser2150Ala). The portion of truncated polypeptide chains after the tandem purification strategy was quantified to roughly 30%. Truncated proteins reflect insufficient amber suppression and are co-purified by heterodimer formation. In-gel fluorescence demonstrates successful labelling with fluorophore BCN-649. (**B**) Purification of three full-length mFAS variants (Gly2113AzPhe, Gly2113AzPhe with Ser2150Ala, and wild type) in preparative 100 µg scale via HiPur Ni-NTA magnetic beads. Samples were clicked with 16 equiv. BCN-649 for 1 h followed by *in vitro* phosphopantetheinylation with CoA and Sfp. In-gel fluorescence of a SDS-PAGE (Bis-Tris 4–12%) was detected before Coomassie-staining. The lowest panel indicates that the majority of free fluorophore is washed away after the first washing step. (**C**) Analysis of the integrity of the three labelled samples after clicking and purification by HPLC-SEC. The main peak at 7.5–7.6 min corresponds to the native dimeric state. Absorbance was monitored at 280 nm and 655 nm and normalized to the highest peak in the UV signal. Both samples containing the ncAA AzPhe were labeled with fluorophore (28% and 31%), whereas the wild type mFAS showed only minor non specific fluorophore binding (5%). (**D**) Activity of mFAS variants monitored by a NADPH consumption assay after phosphopantetheinylation and clicking. The variant Gly2113AzPhe showed one third of the wild type activity, whereas the ACP knock-out (Ser2150Ala) could not produce fatty acids. Error bars indicate the technical repeatability determined from three repeated experiments per construct. (**E**) SDS-PAGE (NuPAGE Bis-Tris 4–12%) of the same three variants: 1: wild type mFAS, 2: mFAS (Gly2113AzPhe and Ser2150Ala) and 3: mFAS (Gly2113AzPhe) after enzymatic labelling with CoA-547 and clicking of the AzPhe containing variants with BCN-649. In-gel fluorescence was detected with three different settings: excitation with green light and filter F595 (for 595 nm); excitation with red light and filter F695 (695 nm) and excitation with green light and filter F695. The gel after Coomassie-staining is attached. All samples show specific fluorescent bands in the respective channels with little unspecific binding due to denaturing conditions. The doubly labelled sample Gly2113AzPhe shows FRET signal. (**F**) Fluorescence analysis of the three labelled samples of (**E**) via HPLC-SEC. Emission spectra are shown for the settings: Ex. 550 nm/Em 570 nm; Ex. 630 nm/Em 650 nm and Ex. 550 nm/Em 650 nm. All signals were normalized as described in detail in the methods section. Again, the doubly labelled sample Gly2113AzPhe shows (highest) FRET signal.

The positions identified to have successful incorporation of ncAAs within the excised ACP domain were tested on the full-length protein. For this, we chose three amber mutation sites and introduced AzPhe at position Gly2113 (Gly01 in ACP) and NorLys2 at position Leu2166 (Leu54 in ACP) as promising candidates, and NorLys2 at position Gln2182 (Gln70 in ACP) as a negative control in mFAS. In agreement with our previous data, the variants Gly2113AzPhe and Leu2166NorLys2 expressed and purified in good yields (66% and 31% of wild type mFAS expression, respectively, for a representative gel of the purification see Fig. 6A), whereas the mutant Gln2182NorLys2 showed poor expression (1% of wild type mFAS expression) (see Supplementary Fig. S7A). Furthermore, it became apparent from western blot analysis (using antibodies against the N-terminal Strep-tag and the C-terminal His-tag) (see Supplementary Fig. S7 B) that amber codon suppression was insufficient, leading to the formation of heterodimers with truncated protein due to the dimeric nature of mFAS, which could not be separated with the current purification methods^43^. These truncated fragments were quantified from band intensities in the gel to be about 30% in the Gly2113AzPhe sample and 40% in the Gln2182NorLys2 sample. The successful incorporation of the respective ncAAs was qualitatively confirmed by clicking complementary fluorophores (see Supplementary Fig. S7C), although also some amount of unspecific binding was observed. The SPAAC reaction was again more efficient than the IEDDAC and yielded interestingly higher in-gel fluorescence intensities than the enzymatic labelling with CoA 647. This may be explained by lower accessibility of the ACP domain in the full-length protein by the complex of Sfp with CoA 647 in an *in vitro* reaction (see Supplementary Fig. S8). We can exclude a partial phosphopantetheinylation  of the enzyme *in vivo* as mFAS is inactive unless co-expressed with Sfp, and hence *E*. *coli* is not capable to post-translationally modify the ACP domain^43^.

We then proceeded with investigating the mutant Gly2113AzPhe in preparative scales. In the first step, a protocol for purification with HisPur Ni-NTA magnetic beads was established to discard excess free fluorophore resulting from the click reaction. About 25–30% of the protein sample was recovered after clicking (Fig. 6B). Three samples were phosphopantetheinylated with Sfp *in vitro* and purified via magnetic beads: mutant Gly2113AzPhe bearing an ACP knock-out (Ser2150Ala) and mutant Gly2113AzPhe, both as protein BCN-POE_3_-NH-DY649P1 conjugate, and wild type mFAS as control. The integrity of all mFAS samples was confirmed by HPLC-SEC (Fig. 6C), demonstrating specific labelling of the AzPhe mutants and minimal non specific binding to wild type mFAS. These three samples were subject to NADPH activity assays, showing no activity before the procedure of activation and clicking, and giving the results depicted in Fig. 6D after removal of excess compounds. The wild type mFAS showed high activity (853 ± 44 nmol/min/mg) revealing that it was not compromised by the clicking conditions and that the mild SPAAC reaction performs appropriate to mFAS modification. As expected, the ACP knock-out sample showed loss of activity, whereas the labelled Gly2113AzPhe variant was active, although the efficiency was reduced (284 ± 57 nmol/min/mg protein). This behaviour may possibly be attributed to truncated proteins within the sample, but this cannot fully explain the complete effect and it further seems that the modification decreases the catalytic efficiency of the enzyme.

As a follow up, we attached two different fluorescent labels to variant Gly2113AzPhe, necessary for FRET experiments. The sample was modified with the BCN-POE_3_-NH-DY649P1 conjugate and enzymatically labelled with CoA 547 by Sfp *in vitro*. Data obtained on this sample indeed showed a FRET signal in denaturing SDS-PAGE (Fig. 6E) and native HPLC-SEC (Fig. 6F) compared to the appropriate control experiments, but nevertheless the DOL was low. Our proposed methods require further optimization to achieve high quality samples for single molecule FRET.

## Discussion

In the last decade, amber codon suppression has emerged as a powerful tool to investigate structure and dynamics of proteins. The opportunity to label site-specifically with bioorthogonal functional groups has led to novel biophysical probes *in vitro* and *in vivo*^19,20,44–46^. So far, this technique has commonly been used to study small proteins, but has not been applied for large and complex multidomain systems.

Conformational variability is a fundamental property for the catalysis of many enzymes and especially megaenzymes such as the animal FAS^6–8,13^. Here, we establish an efficient way to incorporate ncAAs site-specifically with subsequent labelling. Such probes make a range of biological applications accessible, such as photocrosslinking, electron paramagnetic resonance (EPR) spectroscopy, and FRET spectroscopy^44,47,48^. In case of animal FAS, labelled proteins would for example offer the prospect of addressing some of the fundamental questions to carrier domain-mediated substrate shuttling, i.e. towards the time scale of domain-domain interactions, or the influence of loaded intermediates on the mobility of the ACP domain.

**Figure 7:**
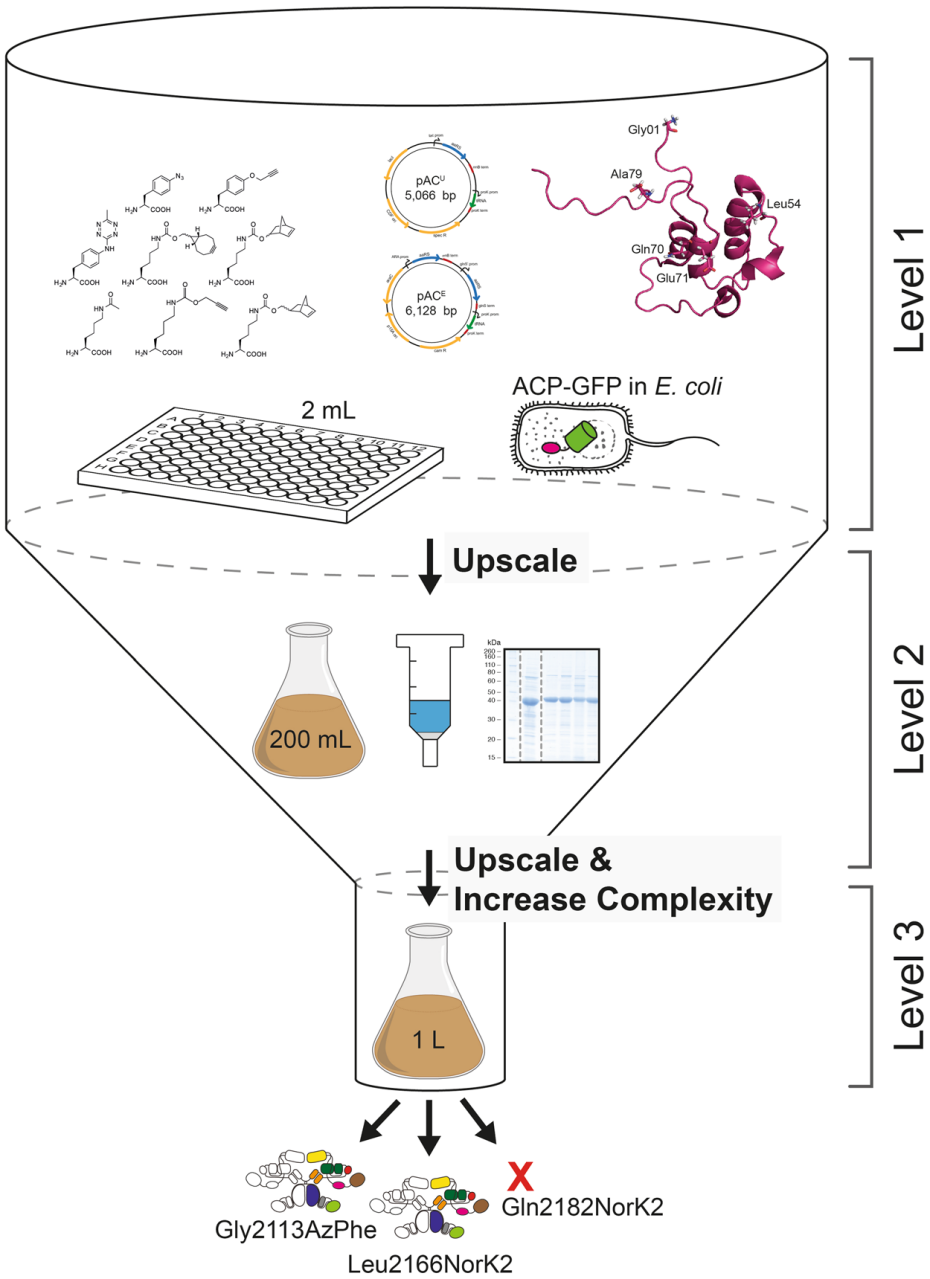
Overview of the workflow of this study. Workflow of amber codon suppression on mFAS divided into three different levels of project progress. Level 1 refers to the low-complex single-domain screening approach in 2 mL small scale cell cultures in 96-well plate format. GFP fluorescence is directly read out and serves as a measure for the efficiency of amber codon suppression. Level 2 refers to the upscaling of culture volumes to 200 mL using initial results from the reporter assay, which also allows obtaining purified protein for further analysis. In a final step, level 3 refers to the application of selected conditions and label positions, that were identified for an individual domain, for the full-length mFAS, being a representative for any comparable multidomain protein.

Recently, we have established recombinant expression and purification of mFAS in *E*. *coli*^43^. We have demonstrated that also parts and single domains of mFAS can be expressed separately, yielding for example the ACP domain as freestanding protein in high yields. Despite this, incorporation of ncAAs by amber codon suppression into such a complex enzyme remains a challenge, owing to the sensitivity of the protein to buffer and temperature changes as well as salt concentrations^49^. To circumvent high consumption of resources, we decided to approach this task by reducing complexity. Specifically, we focused on the small ACP domain, and scaled down expression volumes to 2 mL cultures, which can be conveniently handled and analysed in 96-well format. In order to achieve a fast screening of amber codon suppression conditions, we fused the fluorescent protein GFP C-terminally to the ACP domain. This set-up allows to easily monitor the incorporation of the ncAA by the fluorescence emerging from the full-length fusion construct only. While a universal test system like GFP is typically used to assess general efficiency of amber codon suppression systems, a C-terminal fusion of GFP to a desired domain from a multidomain protein has the advantage of reflecting position dependencies of ncAA incorporation. The initial screening at low complexity was followed by upscaling protocols to eventually make the multidomain protein available as labelled probe (Fig. 7).

Employing this set-up, we have used two reported plasmid systems^26,27^, and compared the incorporation efficiencies across eight different ncAAs, which predominantly allow copper-free click chemistry (Fig. 2A). In our set-up, the two suppressor plasmids pAC^U^ and pAC^E^, derived from pUltra and pEvol vectors, respectively, gave similar performance. The pAC^U^ vector was used for its ease in protein production, as not requiring additional induction of the suppression system along with the protein of interest. In general, the TyrRS from *M*. *jannaschii* performed much better than the PylRS from *M*. *mazei* or *M*. *bakeri*, as the latter suffered from a high tendency to aggregate in *E*. *coli*^50,51^. As the two ncAAs AzPhe and NorLys2 showed optimal incorporation efficiencies of 26–35% and allow copper-free click chemistry, we chose to proceed further with these ncAAs incorporated with the pAC^U^ vector. Another important and practical aspect of these two selected ncAAs is that they can be resourced by well established and easy synthetic routes, which provide the compounds in multigram scale with good yields, but yet at relatively low cost.

In addition to the choice of a suppression system, the reporter assay revealed that the site of ncAA incorporation in the ACP fold was critical for suppression efficiency^40^. Although all amber mutation sites were located on the surface of the ACP domain, to minimize the disturbance of the overall protein fold or any protein-protein interactions in mFAS, both ncAAs were differently sensitive to incorporation sites. Whereas AzPhe was tolerated at all tested sites, NorLys2 was only introduced sufficiently into a disordered loop region. The higher tolerance to incorporate AzPhe at different positions may be explained by its smaller size preserving the integrity of the ACP fold.

Overall, the results of the upscale experiment and the reporter assay agree with one another. Incorporation efficiencies of the two different ncAAs at 5 different mutation sites were in line with data received from the reporter assay, with the only exception of NorLys2 incorporation at site Gly01. From SEC profiles of purified proteins, we were further able to conclude that modification with ncAAs did not disturb the protein fold. With a drop in expression yield to 30–40% of the non-mutated reference construct, also the access to the protein remained satisfyingly high. As a quality control, we finally also employed the constructs in testing bioconjugation with fluorophores. Although there is a discrepancy between the DOL determined using relative in-gel fluorescence intensities and UV-Vis absorbance, both methods agreed that for our set-up the azido-BCN reaction is more efficient than the norbornene-tetrazine reaction.

Application of the identified conditions on the excised ACP domain to the full-length mFAS has led to fluorescently labelled samples that fulfill the criteria of high protein quality, including a maintained oligomeric state and enzymatic activity. Nevertheless, initial experiments have revealed two major issues, which have to be tackled in future studies. The dimeric nature of mFAS, which is an analogous property of structurally related PKSs, has resulted in pairing of truncated polypeptide chains from insufficient suppression of the amber codon with full-length chains. These unwanted heterodimers could not be separated with the applied purification strategy and might interfere in downstream experiments. Although Rangan *et al*. provided protocols to disassemble and reassemble mFAS *in vitro*^49^, owing to protein instability, we are currently working on forming heterodimers *in vivo* by including a second gene encoding mFAS with an alternative C-terminal FLAG-tag in the pAC^U^ vector. This might further provide the opportunity to place fluorescent labels at different positions in both polypeptide chains of the heterodimer. The other important aspect is the relative low degree of fluorescent labelling in the model constructs and even more importantly in the full-length mFAS. The discrepancy in DOL suggests that efficient amber mutation sites may not be as accessible in a multidomain protein, though placed on the surface. But even more importantly, the utilized clickable fluorescent probes in this study, which were chosen due to their commercially availability, suffer from relatively low reaction kinetics^22,23^. A limitation for an enzyme such as mFAS is that it cannot be incubated in click reactions overnight. It is noted that the focus of this field of chemistry is optimizing reactive probes and inventing new interesting compounds^52^.

Our results demonstrate the successful incorporation of ncAAs into a 540 kDa homodimer by amber codon suppression, with subsequent fluorescent labelling. We have devised a systematic approach to tackle the challenges in application of amber codon suppression in an excised small domain instead of the whole multidomain protein, which facilitated the rapid identification of mutation sites. The reporter assay with subsequent upscaling of the culture volume and extending the ncAA modification to the full-length protein allows for possible biophysical methods on such a megaenzyme for the first time. This method led to a minimal resource usage of both chemical and biological samples. The complete approach could be analogously applied to other expressible constructs, as e.g. AT domains or KS-AT didomains. Together, this procedure may be applied to any comparable biological system and can become a powerful tool to elucidate structure and conformational properties of multidomain proteins, as e.g. the homologous PKS family.

## Methods

### Cloning of suppressor plasmids pAC^U^ & pAC^E^

Suppressor plasmids pAC were constructed based on pUltra and pEVOL, as had been published by the lab of Schultz and coworkers^26,27^, by assembling cassettes amplified from the commercially available plasmids pCDF1b, pMAL-c5G and pEVOL_pBpF, which was a gift from Peter Schultz (Addgene plasmid # 31190). Phusion polymerase (Clontech) was used to generate PCR fragments, which were assembled with help of complementary primer overhang in a MegaPrimer PCR and subsequently cloned into the backbone using InFusion Cloning (Takara Bio). The pAC^U^ plasmids encode one copy of aaRS and suppressor tRNA under the tac promoter and rrnB terminator, and the proK promoter and proK terminator, respectively. The backbone of the pAC^U^ contains a CDF origin, spectinomycin resistance and *lacI* gene. The pAC^E^ plasmids encode two copies of aaRS, one under the arabinose promoter and the rrnB terminator, and one under the glnS’ promoter and glnS terminator, as well as one copy of suppressor tRNA under the proK promoter and proK terminator. The backbone of the pAC^E^ contains a p15A origin, chloramphenicol resistance and *araC* gene. Multiple point mutations were introduced to create a set of evolved aaRSs, specific for certain ncAAs. Three different sets of orthogonal suppressor pairs (aaRS/tRNA), derived from *M*. *jannaschii*, *M*. *mazei* and *M*. *barkeri*, are available. Genes of the orthogonal pair mmPylRS/tRNA were obtained from plasmid pJZ, which was a gift from Nediljko Budisa, and mbPylRS was obtained from pAcBac1.tR4-MbPyl, which was a gift from Peter Schultz (Addgene plasmid # 50832). Stellar cells were used for plasmid amplification. All mutations were confirmed by sequencing (Seqlab).

### Cloning of ACP-GFP-fusion constructs and amber mutations

The genes for ACP-GFP fusion constructs were cloned into a pET22b vector, which contains a pBR322 origin, ampicillin resistance and *lacI* gene. They are encoded under a T7 promoter and terminator, and feature a N-terminal Strep-tag and C-terminal His8-tag. The ACP-GFP construct termed wild type in this study contained a Met72Leu mutation, to prevent an alternative translation start and to reduce background GFP-fluorescence. An amber mutation was introduced site-specifically in the wild type gene and its position was varied throughout the ACP sequence to generate six different ACP-GFP mutant constructs, with different incorporation sites.

### General protein expression procedure

All constructs were transformed in *E*. *coli* BL21 Gold (DE3) cells (Agilent Technologies) following the provided protocol. For incorporation of ncAAs, plasmids encoding ACP-GFP and mFAS constructs with amber mutations (for a list of used plasmids, see Supplementary Tables S3 and S4) were co-transformed with the appropriate suppressor plasmid pAC^U^ or pAC^E^. LB agar (Lennox) transformation plates contained 1% glucose to suppress leaky expression, and were supplemented with either 100 µg/mL ampicillin for transformation of the wild type protein, 50 µg/mL ampicillin and 25 µg/mL spectinomycin for co-transformation with pAC^U^ plasmids, or 50 µg/mL ampicillin and 17 µg/mL chloramphenicol for co-transformation with pAC^E^ plasmids. Colonies were grown at 37 °C overnight or at room temperature over weekend and stored at 4 °C up to several weeks. A randomly picked single clone was used to inoculate a pre-culture of Lysogeny Broth, supplemented with 1% glucose and respective antibiotics, which was grown at 37 °C and 180 rpm overnight. The pre-culture was used to inoculate Terrific Broth medium, supplemented with respective antibiotics. The cells were cultivated at 37 °C and 140–180 rpm until an OD_600_ of 0.5–0.7 was reached. The expression culture was supplemented with 2 mM final concentration of the ncAA and expression of the protein constructs was induced with 0.25 mM final concentration of IPTG. Since the genes of pAC^U^ plasmids stand under a tac promoter, no additional induction was needed, whereas expression of the orthogonal suppressor pair from pAC^E^ plasmids was induced additionally with 0.02% final concentration of arabinose. Protein expression was carried out at 20 °C and 140–180 rpm overnight.

### Reporter assay

The reporter assay was performed in 2 mL scale in 96-well deep well plates in technical triplicates, using an ACP-GFP wild type construct without amber mutation as reference and negative samples of each construct without addition of ncAA. The cells were harvested by centrifugation (3,220 rcf for 5 min at 4 °C), washed and resuspended in 300 µL PBS.

### Expression of ACP-GFP constructs

Large scale expression of ACP-GFP constructs was carried out in 200 mL expression cultures. Prior to harvesting the cells by centrifugation (4,000 rcf for 20 min at 4 °C), 2 mL samples of cell cultures were taken for further quantification using GFP-fluorescence. All cell pellets were flash frozen in liquid nitrogen and stored at −80 °C until use.

### Expression of mFAS constructs

Large scale expression of mFAS constructs was carried out in 1 L expression cultures following the protocol by Rittner *et al*.^43^. The cells were harvested by centrifugation (4,000 rcf for 20 min at 4 °C) and subsequently purified.

### Purification of ACP-GFP constructs

The cell pellets were thawed on ice and resuspended in 10 mL His buffer (50 mM KPi, 200 mM NaCl, 20 mM imidazole, 10% glycerol, pH 7.4) containing DNase I and 1 mM EDTA. French pressure cell press was used for mechanical disruption at a pressure of 1000 bar and the cell debris was removed by centrifugation (50,000 rcf for 30 min at 4 °C). After addition of 2 mM MgCl_2_, the lysate was subjected to 3 mL (bead capacity 50 mg/mL) Ni-NTA Superflow resin (QIAGEN) and incubated for 1 h at 4 °C. Unbound protein was washed off with 5 column volumes His buffer and bound His-tagged protein was eluted with 2.5 column volumes His buffer containing 300 mM imidazole and additional 2 column volumes of His buffer with imidazole increased to 500 mM. The elution fractions were analysed by SDS-PAGE and size exclusion chromatography (SEC) over a Superdex 200 Increase 10/300 GL column (His buffer filtered and degassed). Protein samples were concentrated using an Amicon Ultra concentration device (Millipore), flash frozen in liquid nitrogen, and stored at −80 °C.

### Purification of ACP-GFP fluorophore conjugates

Excess fluorophore from bioconjugation reaction was removed by purification over 1 mg HisPur Ni-NTA magnetic beads (Thermo Fisher Scientific). At each purification step the beads were shortly vortexed, spun down and placed in a magnetic stand, so the liquid phase could be taken up with a pipette. The Ni-NTA beads were first equilibrated with 160 µL and additional 400 µL His buffer (50 mM KPi, 200 mM NaCl, 20 mM imidazole, 10% glycerol, pH 7.4). The bioconjugation reaction was diluted with one volume of His buffer and incubated with the Ni-NTA beads for 30 min in the dark on an end-over-end rotator. Unbound protein was washed off with two times 400 µL His buffer. In two elution steps, the bound His-tagged protein was incubated for 30 min, and 15 min respectively, in the dark on an end-over-end rotator with 50 µL His buffer containing 300 mM imidazole.

### Purification of mFAS constructs

The cell pellets were resuspended in 30 mL His buffer (50 mM NaPi, 450 mM NaCl, 10 mM imidazole, 20% glycerol, pH 7.6) containing DNase I and 1 mM EDTA. French pressure cell press was used for mechanical disruption at a pressure of 1000 bar and the cell debris was removed by centrifugation (50,000 rcf for 30 min at 4 °C). After addition of 2 mM MgCl_2_, the protein was bound to Ni-NTA resin (QIAGEN) and eluted at 300 mM imidazole. Additionally to Ni-IMAC the mFAS constructs were purified over a Strep-column (Iba), eluted with 2.5 mM desthiobiotin (Strep buffer: 250 mM KPi, 1 mM EDTA, 1 mM DTT, 10% glycerol, pH 7.4). Further purification was performed by size exclusion chromatography over a Superdex 200 Increase 10/300 GL column (Strep buffer filtered and degassed). Protein samples were concentrated using an Amicon Ultra concentration device (Millipore), flash frozen in liquid nitrogen, and stored at −80 °C.

### Protein concentration

Protein concentrations were calculated from the absorbance at 280 nm, which was recorded on a NanoDrop 2000c (Thermo Scientific). Extinction coefficients were calculated from the primary sequence with CLC Main workbench (Qiagen). Absorbance 1 g/L at 280 nm (10 mm): 0.893 for FAS; 0.558 for ACP-GFP.

### Quantification of GFP-fluorescence

The reporter assay samples and the 2 mL samples from large scale ACP-GFP expression were analysed by their GFP-fluorescence. An undiluted sample or 10–fold dilution of the resuspended cells in PBS was transferred into 96-well plates and OD_600_ and GFP fluorescence was measured at CLARIOstar (BMG). Blank corrected fluorescence values were normalized by OD_600_. Fluorescence intensity of the wild type was set to 100% and all other fluorescence signals were related to the wild type.

### Mass spectrometric protein analysis

Purified protein was analysed using nanoESI (Synapt G2-S) mass spectrometry. Protein buffer of the sample was changed to 0.1 or 1 M ammonium acetate in an Amicon Ultra concentration device (Millipore). Protein concentration of the samples was 1 mg/mL.

### Western blot analysis

For western blot analysis, samples of mFAS constructs were analysed on a SDS-PAGE. The proteins were transferred from the analytical polyacrylamide gel onto a PVDF-membrane by an electrophoretic tank-blot method (25 V for 1 h). The membrane was subsequently blocked with 0.2% I-Block and 0.1% Tween-20 in PBS, treated first with monoclonal mouse anti-Strep antibody (StrepMAB classic, Iba) and monoclonal rabbit anti-His antibody (bethyl) (at 4 °C overnight), and secondly with IgG donkey anti-mouse DyLight 755 and IgG goat anti-rabbit DyLight 633 (Thermo Scientific) (light-protected at room temperature for 1 h). Excess antibodies were washed off in several washing steps with 0.2% I-Block and 0.1% Tween-20 in PBS. The western blot was developed at the Fusion SL Fluorescence Imaging System (Vilber Lourmat) with excitation in the near infrared to infrared range and using the emission filters F-695 Y5 and F-820.

### Fluorescent bioconjugation

Bioconjugation of fluorophore (20–150 equiv.) and protein (1 equiv.) was performed in a copper-free environment at room temperature in the dark. The reaction proceeded in 25–100 µL His buffer for up to 4 hours. Protein mutants with the AzPhe were treated with the fluorophore BCN-POE_3_-NH-DY649P1, and mutants with the NorLys2 were treated with 6-methyl-tetrazine-ATTO-647N. Wild type ACP-GFP served as negative control, treated with the same fluorophores under the same conditions. As reference, 1 equiv. wild type ACP was phosphopantetheinylated with 5 equiv. of the fluorescent substrate CoA 647 (NEB), catalysed by 0.5 equiv. 4′-phosphopantetheinyl transferase Sfp from *B*. *subtilis*. The phosphopantetheinylation was performed in presence of 10 mM MgCl_2_ for 30–45 min at 37 °C in the dark. Subsequent to bioconjugation, an analytical SDS-PAGE was performed and fluorescent protein bands were detected at the Fusion SL Fluorescence Imaging System (Vilber Lourmat) with excitation in the near infrared range and using emission filter F-695 Y5. The FusionCapt Advance Solo 4 16.08a software was used to quantify the fluorescence of the protein bands on the polyacrylamide gel.

### UV-Vis spectra

UV-Vis spectra were recorded on a Carry 100 UV-Vis spectrophotometer (Agilent Technologies) from 800 nm to 220 nm wavelength in quartz glass cuvettes (50 µL sample). Excess fluorophore had been removed by purification over HisPur Ni-NTA magnetic beads (Thermo Fisher Scientific). The reference sample contained His buffer with 300 mM imidazole. Absorption at 650 nm (maximum absorption of the fluorophore) and at 485 nm (maximum absorption of GFP) was used to determine the degree of labelling, following equation ${\rm{DOL}}=\frac{{{\rm{A}}}_{{\rm{\max }}}{{\rm{\varepsilon }}}_{{\rm{GFP}}}}{({{\rm{A}}}_{{\rm{GFP}}}-{{\rm{A}}}_{{\rm{\max }}}{{\rm{CF}}}_{485}){{\rm{\varepsilon }}}_{{\rm{\max }}}}$, with ε_GFP_ and ε_max_ being the molar extinction coefficients of GFP and the fluorophores, respectively. The correction factor ${{\rm{CF}}}_{485}=\frac{{{\rm{A}}}_{485}}{{{\rm{A}}}_{{\rm{\max }}}}$ was determined from the absorption spectrum of the free fluorophore in water. All values for calculation are summarized in Table 1.

**Table 1:** Calculation of the DOL with optical properties of used fluorophores.

Fluorophore	A_GFP_ (mAU)	ε_GFP_ (L mol^−1^ cm^−1^)	A_max_ (mAU)	ε_max_ (L mol^−1^ cm^−1^)	CF_485_
DY647P1	1.09982431	83300	1.92440999	250000	0.006
DY649P1	0.52647227	83300	0.93045866	250000	0.004
ATTO-647N	0.7810781	83300	0.61860055	150000	0.013

### Preparative scale fluorescent bioconjugation and purification of AzPhe labelled FAS

100 µg of purified FAS (2 µM) was labelled in a reaction volume of 100 µL (half Strep and half His buffer without imidazole) enzymatically with Sfp (final concentration: 10 µM MgCl_2_, 12 µM CoA 547 (NEB) and 0.1 µM Sfp) or BCN-POE_3_-NH-DY649P1 (final concentration: 28 µM). Enzymatic labelling was performed for 30 min at 37 °C and followed by SPAAC for 1–2 h at room temperature. The reaction mixture was applied to 100 µL HisPur Ni-NTA magnetic beads suspension (ThermoFisher) and incubated for 15 min on ice. The flow through was again applied to 50 µL magnetic beads to maximize the yield. After 10 min on ice the flow through was discarded (recovery of excess fluorophore is possible), and the beads were resuspended in 200 µL and 100 µL His buffer, respectively. After pooling both samples, the beads were washed twice with 200 µL His buffer and finally eluted with 100 µL His elution buffer (300 mM imidazole). The elution fractions were diluted and concentrated twice with 400 µL Strep buffer in 100 K Amicon Ultra-0.5 mL centrifugal filters to yield 20–30 µg of labelled FAS.

### HPLC-SEC analysis of fluorescently labelled FAS

HPLC analysis of FAS was performed using a Dionex UltiMate 3000 RSLC equipped with a RS fluorescence detector. Chromatographic separation was performed on a SEC column (bioZen 1.8 µm SEC-3) in the buffer (150 mM KPi, pH 6.8). Proteins were detected by monitoring the absorbance at 280 nm, 554 nm and 674 nm or the fluorescence at Em./Ex.: 550/570 nm; 630/650 nm and 550/650 nm. Dimeric FAS was found to elute at 7.5–7.6 min and 7.7–7.8 min when monitored by fluorescence.

Emission spectra of the donor (Ex. 550 nm/Em. 570 nm: 550/570) and acceptor (Ex. 630 nm/Em. 650 nm: 630/650) channels were normalized to the sample with the highest intensity in the respective channel. Additionally, the signal was corrected for differences in the amount of protein by using the absorption at 280 nm. Analysing the channel (Ex. 550 nm/Em. 650 nm: 550/650) was more difficult as the Dy547 fluorophore of CoA-547 shows relatively strong emission in the respective channel falsifying the qualitative FRET signal. Hence, we calculated a correction factor of 0.013 by dividing the fluorescence signal of the CoA-547 labelled mFAS sample at (550/650) by the signal at (550/570). We multiplied both fluorescence signals of AzPhe containing samples at (550/570) with this correction factor to obtain the potential crosstalk of Dy547 in the channel (550/650). This intensity was subtracted from and divided by the signal at (550/650). All signals were multiplied with 0.32 (Gly2113AzPhe) and with 0.59 (Gly2113AzPhe and Ser2150Ala) and than normalized to the highest peak of sample Gly2113AzPhe. Crosstalk of the Dy649P1 fluorophore in channel (550/650) was not considered.

### Overall fatty acid synthase activity

FAS activity was measured by following the oxidation of NADPH at 25 °C in 75 mM potassium phosphate (pH 7.0), 0.5 mM EDTA, 1 mM ascorbic acid, 130 µM acetyl-CoA, 130 µM malonyl-CoA and 80 µM NADPH. The absorbance at 334 nm was monitored with a NanoDrop (cuvette mode) using an extinction coefficient for NADPH of 6220 M^−1^cm^−1^. The enzyme was prepared in a 4-fold stock containing 20% (v/v) PEG 400 for stabilization, resulting in a final protein concentration of 20 nM and 5% (v/v) PEG 400 in the assay. The reaction was initiated by the addition of enzyme to the reaction mixture. Every measurement was performed in technical triplicates and the corresponding background (without enzyme) was subtracted.

## Electronic supplementary material

Supporting Information

## Data Availability

All data generated or analysed during this study are included in this published article and its Supplementary Information files. The plasmids (pAC^U^ and pAC^E^) generated during the current study are available from the corresponding author on reasonable request.
