# Supplementary material for: Site-Specific Labelling of Multidomain Proteins by Amber Codon Suppression
Source: Sci Rep. 2018 Oct 5;8:14864. doi: 10.1038/s41598-018-33115-5 (PMC6173736; doi:10.1038/s41598-018-33115-5)
Supplement: Supplementary file 1 — Supporting Information [file 41598_2018_33115_MOESM1_ESM.pdf]

Supplementary Information

Site-Specific Labelling of Multidomain Proteins by Amber Codon Suppression

Christina S. Heil[+], Alexander Rittner[+], Bjarne Goebel, Daniel Beyer and Martin Grininger*

Author affiliation

Institute of Organic Chemistry and Chemical Biology, Buchmann Institute for Molecular Life Sciences, Cluster of Excellence for Macromolecular Complexes, Goethe University Frankfurt, Max-von-Laue-Str. 15, 60438 Frankfurt am Main, Germany.

Correspondence

*grininger@chemie.uni-frankfurt.de

[+]these authors contributed equally to this work

Table of contents:

Supplementary Methods:

1. Synthesis of non-canonical amino acids
2. Detailed cloning procedure

Supplementary Data:

Mass spectrometric analysis of ACP-GFP constructs

Supplementary Figures:

Figure S1: Cloning scheme of pAC^U and pAC^E vectors
Figure S2: Screening for the most efficient suppression vector system
Figure S3: Testing the effect of different ncAA concentrations
Figure S4: Optimizing the click reaction conditions for fluorescent labelling
Figure S5: Quantification of *in vitro* phosphopantetheinylation
Figure S6: Removal of excess free fluorophore
Figure S7: Production of ncAA-modified mFAS mutants
Figure S8: *In vitro* phosphopantetheinylation of mFAS
Figure S9: Large scale expression and purification of ACP-GFP mutants
Figure S10: Fluorescent labelling of ACP-GFP mutants

Supplementary Tables:

Table S1: List of primers

Table S2: List of amber codon suppressor plasmids

Table S3: List of expression plasmids for ACP-GFP constructs

Table S4: List of expression plasmids for mFAS constructs

Supplementary Methods

1. Synthesis of non-canonical amino acids

All chemicals were used as purchased from Sigma-Aldrich without further processing. Protected amino acids were ordered at Iris Biotech. All syntheses except for AzPhe were performed under dry conditions and argon atmosphere.

1.1. Synthesis of AzPhe (**2**)[1]

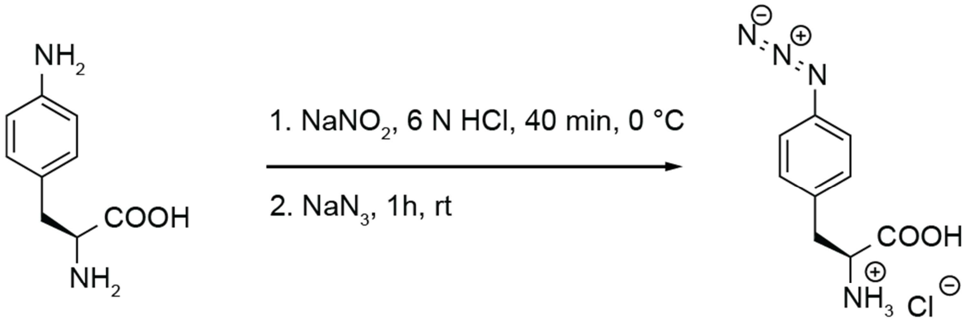

1 **2**

4-Aminophenylalanine **1** (7.2 g, 34.7 mmol, 1.0 equiv.) was dissolved in 6 N HCl solution (20 mL) and cooled down to 0 °C. Subsequently, a solution of $NaNO_2$ (2.8 g, 40.5 mmol, 1.2 equiv.) in water (5 mL) was prepared and slowly added. The reaction mixture was diluted with 6 N HCl solution (33 mL) and further stirred for 20 min at 0 °C. Then, a solution of NaN_3 (2.5 g, 38.5 mmol, 1.1 equiv.) in water (6 mL) was prepared and added slowly. The reaction mixture was kept at 0 °C for another 15 min and allowed to warm to room temperature for 1 h. The hydrochloride was filtered and washed with cold diethyl ether. Recrystallization with absolute ethanol and three volumes of diethyl ether over night at –20 °C afforded **2** as pale yellow solid (7.3 g, 81%).

^{1}H-NMR (250 MHz, DMSO-d_6) δ (ppm) = 7.34 (d, ^3J = 8.4 Hz, 2H), 7.10 (d, ^3J = 8.4 Hz, 2H), 4.13 (dd, ^3J = 6.4 Hz, 6.3 Hz, 1H), 3.17-3.03 (m, 2H).

IR (KBr): Azide band at 2140 cm^{-1} matches with the literature[2].

1.2. Synthesis of PrPhe (**6**)

1.2.1. Synthesis of methyl-2-(*tert*-butoxycarbonyl)amino)-3-(4-(ethynyloxy)phenyl)propanoate **4**[3]

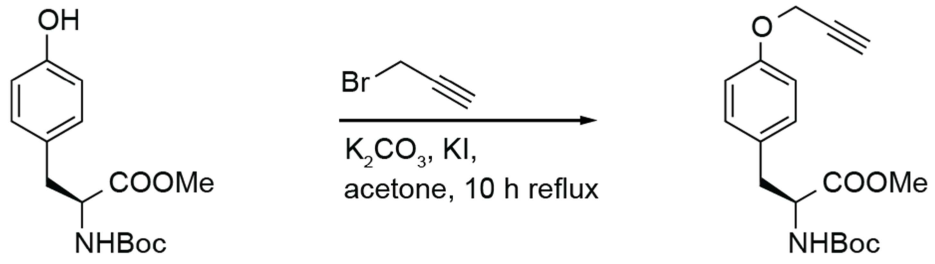

3 **4**

K_2CO_3 (9.10 g, 67.5 mmol, 1.5 equiv.) and KI (0.72 g, 4.50 mmol, 0.1 equiv.) were added to a stirred solution of Boc-L-Tyr-OMe **3** (13.1 g, 45.0 mmol, 1.0 equiv.) in acetone (150 mL) at room temperature. Propargyl bromide (6 mL, 45 mmol, 1 equiv.) was added dropwise over 30 min and the resulting mixture was heated under reflux for 10 h. The reaction mixture was cooled on ice and filtered two times. The filtrate was concentrated *in vacuo* and the residue was re-dissolved in ethyl acetate (100 mL), washed twice with water (30 mL), and dried over $MgSO_4$. Evaporation of the solvent *in vacuo* afforded **4** as yellow oil (14.1 g, 94%).

^{1}H-NMR (300 MHz, $CDCl_3$): δ (ppm) = 7.06 (d, ^3J = 8.75 Hz, 2H), 6.91 (d, ^3J = 8.75 Hz, 2H), 4.97-4.96 (m, 1H), 4.67 (d, ^3J = 2.50 Hz, 2H), 4.54-4.53 (m, 1H), 3.71 (s, 3H), 3.11-3.03 (m, 1H), 2.53-2.52 (m, 1H), 1.42 (s, 9H).

1.2.2. Synthesis of 2-((*tert*-butoxycarbonyl)amino)-3-(4-(ethynyloxy)phenyl)propanoic acid **5**[4]

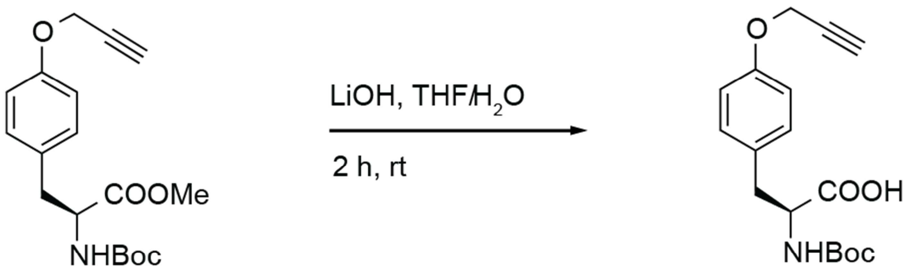

4 **5**

The propargyl derivative Boc-L-PrPhe-OMe **4** (13.3 g, 40.0 mmol, 1.0 equiv.) was dissolved in THF (100 mL) and 1 M LiOH solution (85 mL, 85 mmol, 2.0 equiv.) was added carefully to the stirred solution at room temperature. After 1 h, completion of the reaction was checked by TLC (1:1 EtOAC/n-Hexan, $R_f = 0.8$). The reaction mixture was acidified with conc. HCl (10 mL) and the two phases were separated. The aqueous phase was extracted three times with ethyl acetate and the combined organic phases were washed with brine, and dried over $MgSO_4$. Evaporation of the solvent *in vacuo* afforded **5** as yellow oil (11.7 g, 91%).

^{1}H-NMR (250 MHz, $CDCl_3$): δ (ppm) = 7.13 (d, ^3J = 8.51 Hz, 2H), 6.92 (d, ^3J = 8.70 Hz, 2H), 4.68 (d, ^3J = 2.40 Hz, 2H), 3.19-2.90 (m, 2H), 2.52 (t, ^3J = 2.40 Hz, 1H), 2.05 (s, 1H), 1.43 (s, 9H).

1.2.3. Synthesis of 2-amino-3-(4-(ethynyloxy)phenyl)propanoic acid hydrochloride **6**[5]

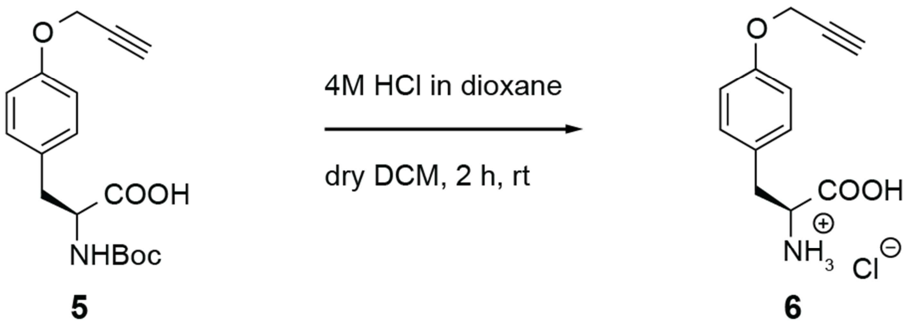

The carboxylic acid Boc-L-PrPhe **5** (11.7 g, 37.0 mmol, 1.0 equiv.) was dissolved in DCM (25 mL) and 4 M HCl in dioxane (15 mL, 60 mmol, 1.6 equiv.) were added to the stirred solution at room temperature. After 2.5 h of reaction, the solvent was evaporated *in vacuo* to afford **6** as colourless solid (9.33 g, 99%).

^{1}H-NMR (400 MHz, DMSO-d$_6$): δ (ppm) = 8.40 (br s, 2H), 7.21 (d, ^{3}J = 8.56 Hz, 2H), 6.94 (d, ^{3}J = 8.56 Hz, 2H), 4.77 (d, ^{3}J = 2.32 Hz, 2H), 4.11-4.08 (m, 1H), 3.57-3.56 (m, 1H), 3.08 (d, ^{3}J = 6.11 Hz, 2H).

MALDI-MS (m/z): $[M+Na]^+$ calcd for $C_{12}H_{13}NO_3$ 242.07876, meas. 242.07914.

1.3. Synthesis of TetPhe (**12**)

1.3.1. Synthesis of methylthiocarbohydrazide **8**[5]

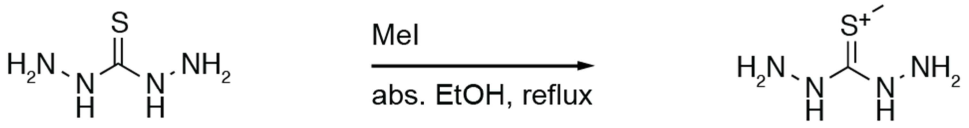

7 **8**

Thiocarbohydrazide **7** (24.1 g, 0.23 mol, 1.0 equiv.) was suspended in abs. ethanol (800 mL) and heated under reflux. Subsequently, iodomethane (16 mL, 0.3 mol, 1.1 equiv.) was added and heating was continued for 1 h. The solution was filtered and stirred for 1 h at room temperature, then kept at 4 ℃ overnight. The solvent was removed by decantin g and remaining solvent was evaporated *in vacuo* to afford **8** as colourless needles (33.3 g, 59%).

^{1}H-NMR (250 MHz, DMSO-d$_6$): δ (ppm) = 2.38 (s, 3H).

1.3.2. Synthesis of 3-methyl-6-(methylthio)-1,2,4,5-tetrazine **9**[6]

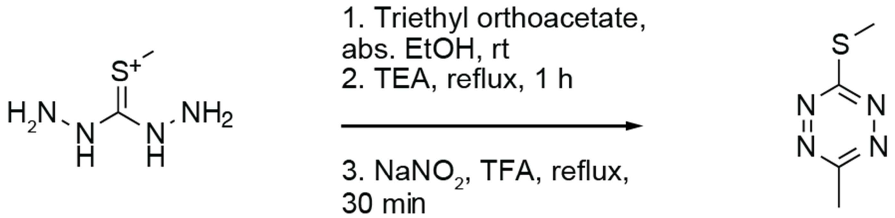

8 **9**

Triethyl orthoacetate (11 mL, 62 mmol, 1.4 equiv.) was added to a stirred suspension of methylthiocarbohydrazide **8** (11.2 g, 45.1 mmol, 1.0 equiv.) in abs. ethanol (300 mL) at room temperature. After 30 min, triethylamine (6.3 mL, 45 mmol, 1.0 equiv.) was added and the mixture was heated under reflux for 1 h. Subsequently, $NaNO_2$ (6.40 g, 92.8 mmol, 2.1 equiv.) and TFA (3.5 mL, 45 mmol, 1.0 equiv.) were added and heating was continued for 30 min. After addition of *n*-hexane (300 mL), the solution was degassed with Ar and stirred for 30 min at room temperature. Then, water (600 mL) was added, and the solution was extracted three times with diethyl ether (300 mL), and dried over $MgSO_4$. The solvent was evaporated *in vacuo*, and the crude

product was purified on silica gel (19:1 *n*-hexane/DCM) to afford **9** as red oil (1.50 g, 23%).

^{1}H-NMR (250 MHz, $CDCl_3$): δ (ppm) 2.97 (s, 3H), 2.72 (s, 3H).

1.3.3. Synthesis of 2-((*tert*-butoxycarbonyl)amino)-3-(4-((6-methyl-1,2,4,5,-tetrazine-3-yl)amino)phenyl)propanoic acid **11**[5]

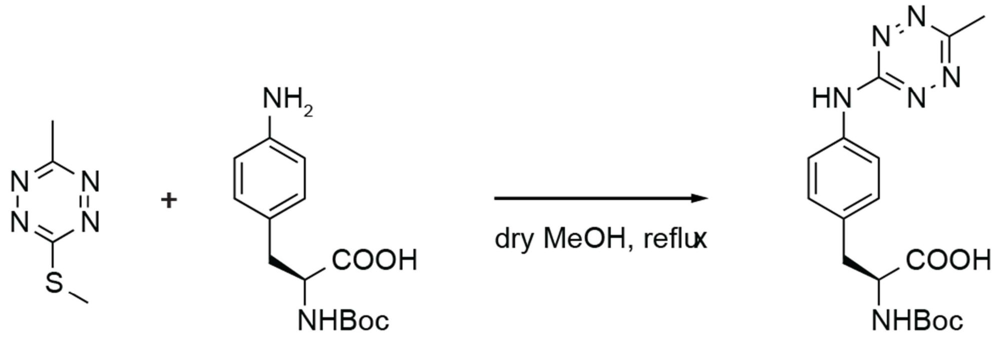

9 **10** **11**

The tetrazine derivative **9** (0.57 g, 4.00 mmol, 1.1 equiv.) and Boc-D-4-aminophenylalanine **10** (1.02 g, 3.60 mmol, 1.0 equiv.) were suspended in methanol (10 mL) and heated under reflux for 18 h. The solvent was evaporated *in vacuo,* and the crude product was purified on silica gel (95:5 $CHCl_3$/methanol) to afford **11** as orange crystals (0.36 g, 23%).

^{1}H-NMR (250 MHz, DMSO-d_6): δ (ppm) = 12.60 (s, 1H), 10.62 (s, 1H), 7.63 (d, ^{3}J = 7.5 Hz, 2H), 7.25 (d, ^{3}J = 7.5 Hz, 2H), 4.08-4.05 (m, 1H), 3.04-2.79 (m, 2H), 2.78 (s, 3H), 1.34 (s, 9H).

1.3.4. Synthesis of 2-amino-3-(4-((6-methyl-1,2,4,5-tetrazine-3-yl)amino)phenyl)propanoic acid hydrochloride **12**[5]

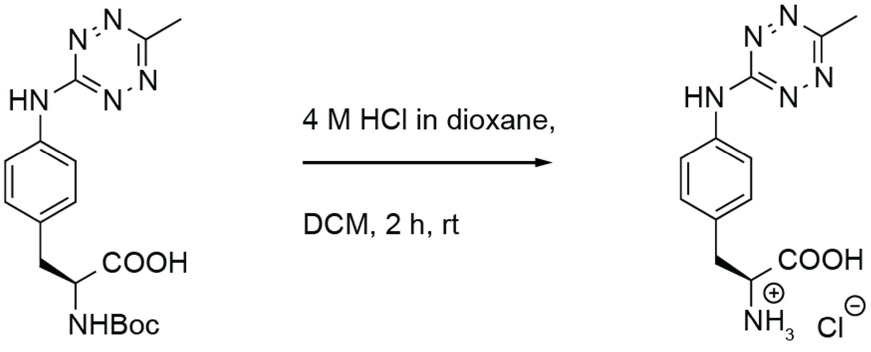

11 **12**

The Boc-protected tetrazine derivative **11** (225 mg, 1.58 mmol, 1.0 equiv.) was dissolved in DCM (8 mL), and 4 M HCl in dioxane (8.0 mL, 32 mmol, 20 equiv.) was added. The solution was stirred for 2 h at room temperature. The solvent was evaporated *in vacuo* to afford **12** as red crystals (161 mg, 87%).

^{1}H-NMR (400 MHz, DMSO-d$_6$): δ (ppm) = 10.70 (s, 1H), 8.41 (br s, 2H), 7.67 (d, ^3J = 8.56 Hz, 2H), 7.28 (d, ^3J = 8.56 Hz, 2H), 4.14 (br t, ^3J = 6.30 Hz, 1H), 3.11 (d, ^3J = 6.30 Hz, 2H), 2.78 (s, 3H).

MALDI-MS (m/z): $[M+Na]^+$ calcd for $C_{12}H_{14}N_6O_2$ 297.10704, meas. 297.10681.

1.4. Synthesis of AcLys (**15**)

1.4.1. Synthesis of 6-acetamido2-((*tert*-butoxycarbonyl)amino)hexanoic acid **14**

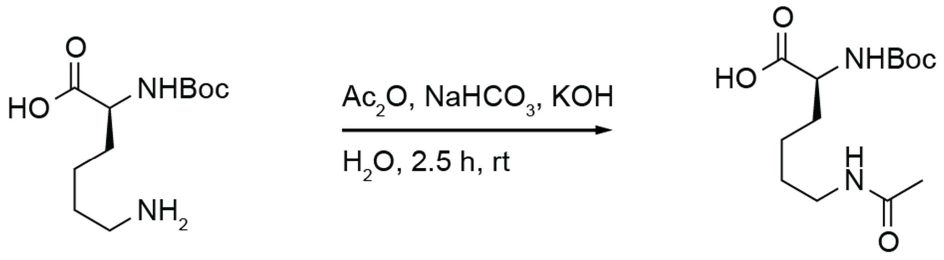

13 **14**

To a solution of Boc-L-lysine **13** (3.06 g, 12.2 mmol, 1.0 equiv.) in water (60 mL), $NaHCO_3$ (3.12 g, 36.6 mmol, 3.0 equiv.) and KOH (0.85 g, 12.2 mmol, 1.0 equiv.) were added. Subsequently, acetic anhydride (1.2 mL, 12 mmol, 1.0 equiv.) was added dropwise over 10 min and the solution was stirred for 2.5 h at room temperature. After addition of ethyl acetate (60 mL), the aqueous phase was acidified with conc. HCl to pH 2. The two phases were separated, and the aqueous phase was extracted three times with ethyl acetate (30 mL). The organic phases were combined, washed with brine and dried over $MgSO_4$. Evaporation of the solvent *in vacuo* afforded **14** as slightly yellow oil, which was used directly in the next step without further purification.

^{1}H-NMR (250 MHz, DMSO-d_6) δ (ppm) = 12.11 (s, 2H), 7.79 (t, ^3J = 5.4 Hz, 1H), 7.01 (d, ^3J = 8.2 Hz, 1H), 3.89-3.77 (m, 1H), 3.00 (q, ^3J = 5.8 Hz, 2H), 2.10 (s, 2H), 1.78 (s, 2H), 1.71-1.47 (m, 2H), 1.39 (s, 9H).

1.4.2. Synthesis of 6-acetamido-2-aminohexanoic acid hydrochloride **15**[5]

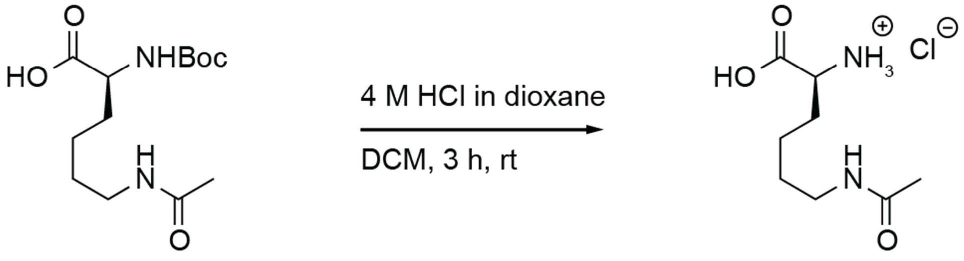

14 **15**

A solution of 6-acetylamino-Boc-L-lysine **14** in dioxane (38 mL) and 4 M HCl in dioxane (3 mL, 12 mmol, 1.0 equiv.) was stirred for 3 h at room temperature. After evaporation of the solvent *in vacuo*, the residue was re-dissolved in 1 M HCl (60 mL). Subsequently, the solvent was removed azeotrope *in vacuo* by addition of toluene (4×25 mL) to afford a yellow oil. Lyophilisation obtained **15** as an off-white powder (2.75 g, 100%).

^{1}H-NMR (250 MHz, DMSO-d_6) δ (ppm) = 8.54-8.47 (m, 2H), 8.06-8.03 (m, 1H), 3.81 (q, ^{3}J = 5.5 Hz, 1H), 3.03-2.96 (m, 2H), 1.79 (s, 5H), 1.45-1.27 (m, 4H).

ESI-MS (m/z): $[M+H]^+$ calcd for $C_8H_{16}N_2O_3$ 189.12, meas. 189.12.

1.5. Synthesis of PrLys (**17)**

1.5.1. Synthesis of 2-((*tert*-butoxycarbonyl)amino)-6-(((prop-2-yn-1-yloxy)carbonyl)amino)hexanoic acid **16**[7]

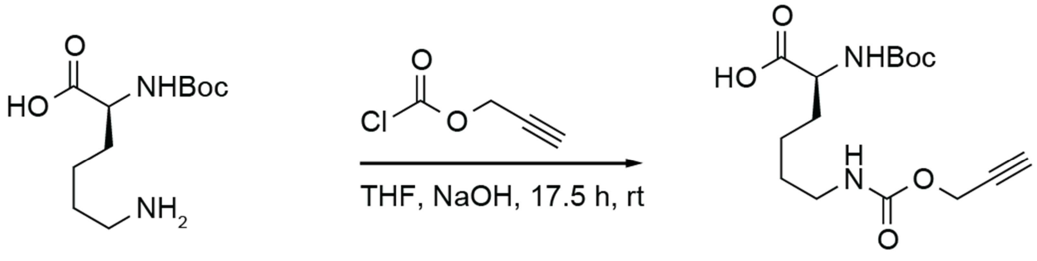

13 **16**

A solution of Boc-L-lysine **13** (3.00 g, 12.2 mmol, 1.3 equiv.) in THF (30 mL) and 1 M NaOH (30 mL) was cooled down to 0 ℃. Subse quently, propargyl chloroformate (1.0 mL, 9.7 mmol, 1.0 equiv.) was added dropwise over 10 min. The solution was stirred at room temperature for 17.5 h. After addition of ethyl acetate (60 mL), the aqueous phase was acidified with conc. HCl to pH 1.5. The two phases were separated, and the aqueous phase was extracted ethyl acetate (30 mL). The organic phases were combined, washed with brine and dried over $MgSO_4$. Evaporation of the solvent *in vacuo* afforded **16** as yellow oil, which was used directly in the next step without further purification.

^{1}H-NMR (400 MHz, DMSO-d_6): δ (ppm) = 12.38 (s, 1H), 7.30 (t, ^3J = 4.3 Hz, 1H), 7.00 (d, ^3J = 7.7 Hz, 1H), 4.80 (d, ^3J = 2.4 Hz, 1H), 4.59 (d, ^3J = 2.2 Hz, 2H), 3.86-3.77 (m, 1H), 3.46-3.43 (m, 1H), 2.96 (q, ^3J = 5.9 Hz, 2H), 1.38 (s, 9H), 1.68-1.46 (m, 2H), 1.33-1.24 (m, 2H).

1.5.2. Synthesis of 2-amino-6-(((prop-2-yn-1-yloxy)carbonyl)amino)hexanoic acid hydrochloride **17**[5]

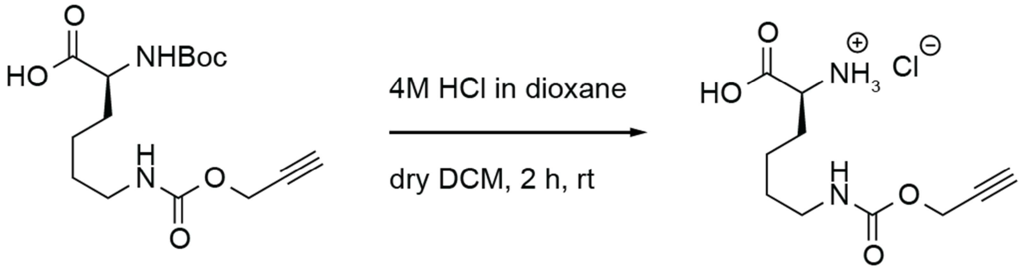

16 **17**

A solution of 6-propargylformylamino-Boc-L-lysine **16** in dioxane (30 mL) and 4 M HCl in dioxane (2.4 mL, 9.6 mmol, 0.8 equiv.) was stirred for 4 h at room temperature. After evaporation of the solvent *in vacuo*, the residue was re-dissolved in 1 M HCl (80 mL). Subsequently, the solvent was removed azeotrope *in vacuo* by three times addition of toluene (25 mL) to afford **17** as an off-white powder (1.70 g, 55.0%).

^{1}H-NMR (400 MHz, DMSO-d$_6$): δ (ppm) = 8.40 (br s, 2H), 7.36 (t, ^3J = 5.44 Hz, 1H), 4.60 (d, ^3J = 2.32 Hz, 2H), 3.85-3.84 (m, 1H), 3.48 (t, ^3J = 2.32 Hz, 1H), 2.98 (q, ^3J = 6.05 Hz, 2H), 1.79-1.78 (m, 2H), 1.41-1.38 (m, 3H).

MALDI-MS (m/z): $[M+Na]^+$ calcd for $C_{10}H_{16}N_2O_4$ 251.10023, meas. 251.10030.

1.6. Synthesis of NorLys1 (**22)**

1.6.1. Synthesis of bicyclo[2.2.1]hept-5-en-2-ol **19**[8]

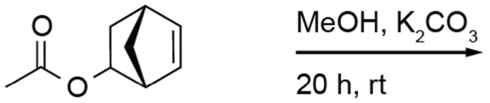

18 **19**

A mixture of 5-norbornene-2-yl acetate **18** (10.0 g, 65.7 mmol, 1.0 equiv.) and K_2CO_3 (9.60 g, 69.7 mmol, 1.1 equiv.) in methanol (100 mL) was stirred for 20 h at room temperature. After evaporation of the solvent *in vacuo*, the residue was re-dissolved in water (300 mL), and extracted with ethyl acetate (5×100 mL). Evaporation of the solvent *in vacuo* afforded **19** as off-white crystals (6.20 g, 85.7%).

^{1}H-NMR (400 MHz, $CDCl_3$) δ (ppm) = 6.46-6.45 (m, 1H), 6.07-6.06 (m, 1H), 4.48-4.47 (m, 1H), 3.00 (s, 1H), 2.82 (s, 1H), 2.14-2.10 (m, 1H), 1.33-1.28 (m, 3H).

1.6.2. Synthesis of bicyclo[2.2.1]hept-5-en-2-yl 2-(2,5-dioxopyrrolidin-1-yl)acetate **20**[9]

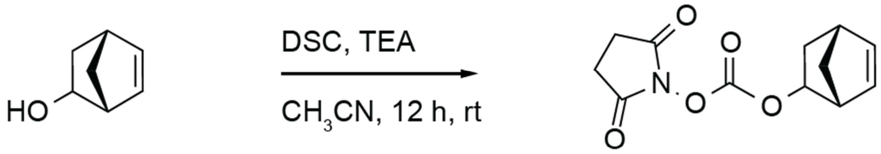

19 **20**

To a solution of the norbornene derivative **19** (3.0 g, 27 mmol, 1.0 equiv.) in acetonitrile (100 mL), triethylamine (12 mL, 87 mmol, 3.2 equiv.) and *N*,*N'*-disuccinimidyl carbonate (11.3 g, 44.0 mmol, 1.2 equiv.) were added, and the mixture was stirred for 12 h at room temperature. After evaporation of the solvent *in vacuo*, the crude product was purified on silica gel (99.5:0.5 DCM/Et_2O) to afford **20** as slightly yellow crystals (6.20 g, 92.0%).

^{1}H-NMR (250 MHz, $CDCl_3$) δ (ppm) = 6.41-6.30 (m, 1H), 6.04-5.94 (m, 1H), 5.36 and 4.73 (m, 1H), 3.27 and 3.08 (m, 1H), 2.91 (s, 1H), 2.82 (s, 4H), 2.24-2.14 (m, 1H), 1.68-1.10 (m, 3H).

1.6.3. Synthesis of 6-(((bicyclo[2.2.1]hept-5-en-2-yloxy)carbonyl)amino)-2-((tert-butoxycarbonyl)amino)hexanoic acid **21**[9]

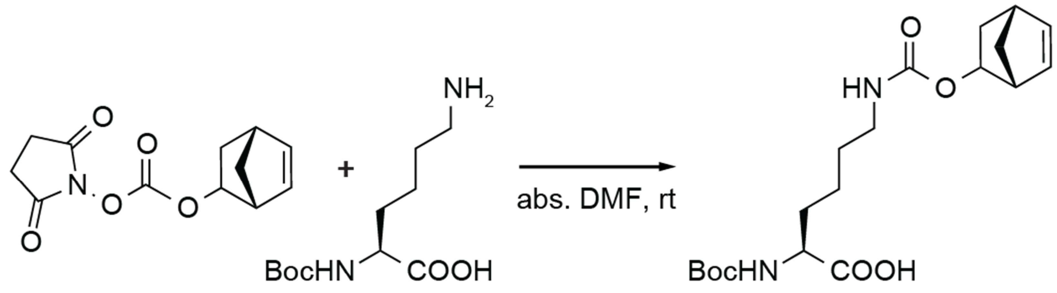

20 **13** **21**

To a solution of the activated norbornene **20** (2.50 g, 10.0 mmol, 1.0 equiv.) in DMF (35 mL), the Boc-protected amino acid Boc-Lys-OH **13** (3.20 g, 13.0 mmol, 1.3 equiv.) was added, and the mixture was stirred over night at room temperature. After addition of water (300 mL), the mixture was extracted with ethyl acetate (150 mL). The organic phases were combined, washed with water (150 mL) and once with brine (75 mL), and dried over $MgSO_4$. Evaporation of the solvent *in vacuo* afforded **21** as white foam, which was used directly in the next step without further purification.

^{1}H-NMR (400 MHz, $CDCl_3$) δ (ppm) = 6.29-6.19 (m, 1H), 5.95-5.93 (m, 1H), 5.30-5.22 (m, 2H), 4.93-4.09 (m, 2H), 3.11 (br s, 2H), 2.80-2.79 (m, 1H), 2.09-2.08 (m, 1H), 1.82-1.30 (m, 15H), 0.88-0.87 (m, 1H).

1.6.4. Synthesis of 2-amino-6-(((bicyclo[2.2.1]hept-5-en-2-yloxy)carbonyl)amino)hexanoic acid hydrochloride **22**[5]

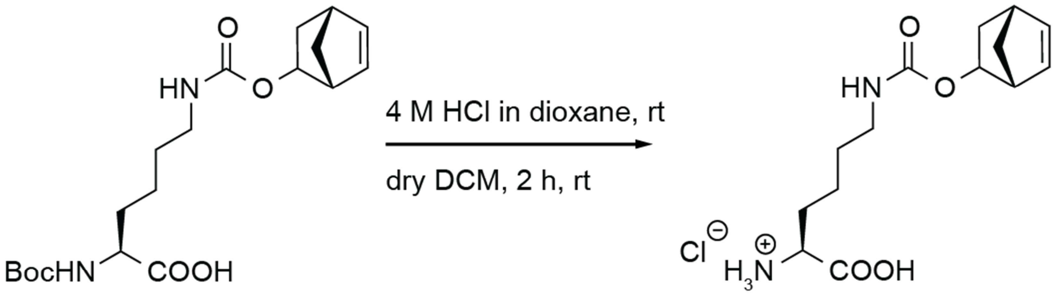

21 **22**

A solution of the Boc-protected norbornene lysine **21** in DCM (25 mL) and 4 M HCl in dioxane (20 mL, 80 mmol, 8 equiv.) was stirred for 1 h at room temperature. After evaporation of the solvent *in vacuo*, the residue was taken up in diethyl ether and filtrated, to afford **22** as colourless solid (3.10 g, 97%).

^{1}H-NMR(400 MHz, DMSO-d_6) δ (ppm) = 7.08-6.91 (m, 1H), 6.31-6.24 (m, 1H), 6.00-5.92 (m, 1H), 5.11-5.07 and 4.44-4.42 (m, 1H), 3.86-3.78 (m, 1H), 3.45-3.27 (m, 1H), 3.03 (s, 1H), 2.97-2.88 (m, 2H), 2.81-2.77 (m, 1H), 2.06-2.00 (m, 1H), 1.77-1.75 (m, 2H), 1.59-1.55 (m, 1H), 1.42-1.29 (m, 5H), 0.80-0.77 (m, 1H).

MALDI-MS (m/z): $[M+Na]^+$ calcd for $C_{14}H_{22}N_2O_4$ 305.14718, meas. 305.14741.

1.7. Synthesis of NorLys2 (**26**)

1.7.1. Synthesis of bicyclo[2.2.1]hept-5-en-2-ylmethyl (2,5-dioxopyrrolidin-1-yl) carbonate **24**[9,10]

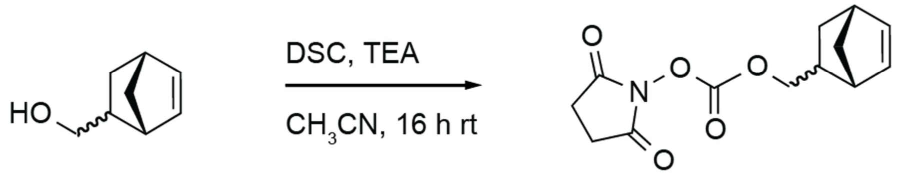

23 **24**

To a solution of *N,N'*-disuccinimidyl carbonate (13.6 g, 53.6 mmol, 1.1 equiv.) and 5-norbornene-2-methanol **23** (5.8 mL, 48 mmol, 1.0 equiv.) in acetonitrile (120 mL), triethylamine (24 mL) were added, and the mixture was stirred at room temperature overnight. After evaporation of the solvent *in vacuo*, the crude product was purified on silica gel (99.5:0.5. DCM/Et_2O) to afford **24** as colourless solid (10.2 g, 80%).

^{1}H-NMR (500 MHz, $CDCl_3$) δ (ppm) = 6.20 (dd, ^3J = 3.1 Hz, 5.7 Hz, 1H), 6.13-6.10 (m, 1H), 5.99 (dd, ^3J = 2.9 Hz, 5.7 Hz, 1H), 4.41 (dd, ^3J = 6.5 Hz, 6.4 Hz, 0.5H), 4.23 (m, 0.5H), 4.11 (dd, ^3J = 6.6 Hz, 10.3 Hz, 1H), 3.94 (dd, ^3J = 9.8 Hz, 10.1 Hz, 1H), 2.97 (s, 1H), 2.55-2.49 (m, 1H), 1.92-1.83 (m, 2H), 1.50 (dd, ^3J = 2.08 Hz, 8.4 Hz, 1H), 1.42-1.18 (m, 3H), 0.61-0.57 (m, 1H).

ESI-MS (m/z): $[M+Na]^+$ calcd for $C_{13}H_{15}NO_5$ 288.25, meas. 288.16.

1.7.2. Synthesis of 6-(((bicyclo[2.2.1]hept-5-en-2-ylmethoxy)carbonyl)amino)-2-((tert-butoxycarbonyl) amino)hexanoic acid **25**[9,10]

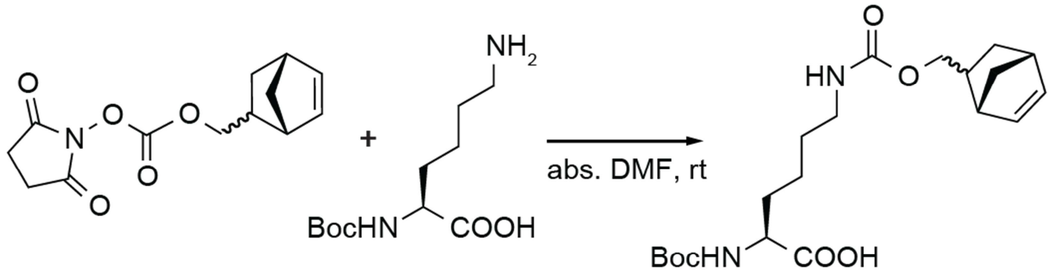

24 **13** **25**

A solution of compound **24** (9.66 g, 36.4 mmol, 1.0 equiv.) and Boc-protected lysine **13** (9.88 g, 40.0 mmol, 1.1 equiv.) in DMF (100 mL) was stirred at room temperature overnight. After addition of water (400 mL), the mixture was extracted with ethyl acetate (500 mL). The organic phase was washed with water (150 mL) and subsequently, the aqueous phase was extracted twice with ethyl acetate (400 mL). The organic phases were combined and washed again with water (400 mL) and brine (200 mL), and dried over $MgSO_4$. Evaporation of the solvent *in vacuo* afforded **25** as slightly yellow foam (14.4 g, 99%).

^{1}H-NMR (500 MHz, $CDCl_3$) δ (ppm) = 6.17-12 (m, 1H), 6.11-6.06 (m, 1H), 5.95-5.94 (m, 1H), 5.29-5.27 (m, 1H), 4.36-4.26 (m, 1H), 3.20-3.17 (m, 3H), 2.98 (s, 1H), 2.89-2.85 (m, 1H), 1.92-1.80 (m, 3H), 1.57-1.51 (m, 3H), 1.48-1.39 (m, 14H).

ESI-MS (m/z): $[M+H]^+$ calcd for $C_{20}H_{32}N_2O_6$ 397.48, meas. 397.25.

1.7.3. Synthesis of 2-amino-6-(((bicyclo[2.2.1]hept-5-en-2-ylmethoxy)carbonyl)amino)hexanoic acid hydrochloride **26**[5]

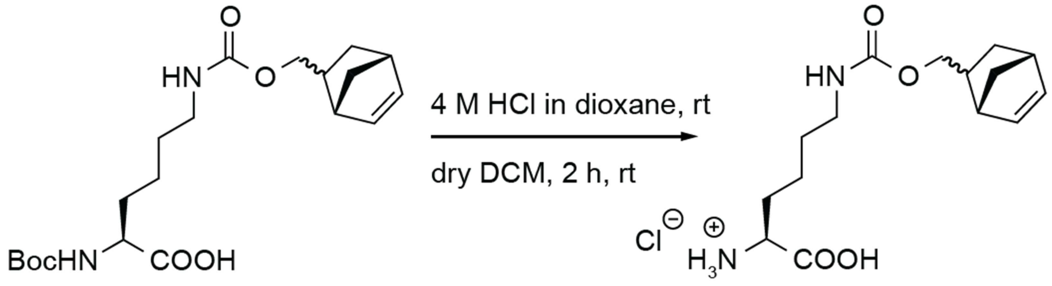

25 **26**

To a solution of the Boc-protected intermediate **25** (14.4 g, 36.0 mmol, 1.0 equiv.) in DCM (100 mL), 4 M HCl in dioxane (80.0 mL, 0.32 mol, 8.8 equiv.) were added and the solution was stirred for 1 h at room temperature. Evaporation of the solvent *in vacuo* afforded **26** as colourless solid (10.4 g, 87%).

^{1}H-NMR (250 MHz, DSMO-d_6) δ (ppm) = 8.31 (m, 2H), 7.14-7.06 (m, 1H), 6.20-6.17 (m, 1H), 6.11-6.09 (m, 1H), 5.95-5.93 (m, 1H), 4.38-4.34 (m, 0.3H), 4.07-4.01 (m, 1H), 2.90 (s, 0.7H), 2.82-2.80 (m, 2H), 2.25-2.23 (m, 1H), 1.86-1.70 (m, 8H), 1.16-1.10 (m, 1H).

MS-ESI (m/z): $[M+H]^+$ calcd for $C_{15}H_{24}N_2O_4$ 297.36, meas. 297.20.

2. Detailed cloning procedure

2.1. Cloning of pAC^U vectors

The pAC^U vector was assembled from cassettes amplified from different commercially available vectors (see Supplementary Fig. S1). The backbone derived from the pCDF-1b vector (EMD Millipore, Novagen), and was cloned as insert with primers CH02 & CH03. The tac promoter and rrnB terminator region derived from the pMAL-c5G vector (NEB, discontinued), and were cloned as inserts with primers CH04 (with 5'-overhang to the pCDF-1b backbone insert) & CH05 and CH32 & CH19.

2.1.1. *Methanococcus jannaschii* derived TyrRS-$tRNA^{Tyr}$ pair

The genes encoding mjTyrRS and $tRNA^{Tyr}$ were cloned as inserts from pEVOL-pBpF with primers CH06 (with 5'-overhang to the tac promoter region from pMAL-c5G) & CH33 (with 5'-overhang to the rrnB terminator region from pMAL-c5G), and CH34 (with 5'-overhang to the rrnB terminator region from pMAL-c5G) & CH11 (with 5'-overhang to the pCDF-1b backbone insert). The vector pEVOL-pBpF was a gift from Peter Schultz (Addgene plasmid # 31190) and contains genes encoding an optimized $tRNA^{Tyr}$ and an evolved mjTyrRS for the incorporation of benzoylphenylalanine. Phusion polymerase (Clontech) was used to generate PCR fragments, which were assembled with help of complementary primer overhang in a MegaPrimer PCR and subsequently cloned into the backbone using InFusion Cloning (Takara Bio). The resulting vector pAC^U_Bpa was used as template for multiple point mutations in the gene of *M. jannaschii* derived tyrosyl-tRNA synthetase to generate a variety of pAC^U vectors encoding evolved mjTyrRSs, specific for incorporation of various substituted phenylalanine ncAAs.

2.1.2. *Methanosarcina mazei/ barkeri* derived PylRS-$tRNA^{Pyl}$ pair

In order to incorporate lysine-derivative ncAAs, a *M. mazei* or *M. barkeri* derived pyrrolysyl-tRNA synthetase and corresponding $tRNA^{Pyl}$ is used. Therefore, the genes encoding mjTyrRS and $tRNA^{Tyr}$ in the pAC^U_Bpa vector were exchanged by genes encoding mmPylRS/mbPylRS and $tRNA^{Pyl}$. The

insert between the genes of PylRS and $tRNA^{Pyl}$ was cloned from pAC^{U} with primers CH32 & AR366 and contained the rrnB terminator and proK promoter sequences. The pAC^{U} backbone was cloned with primers AR369 & CH05.

The inserts encoding mmPylRS and $tRNA^{Pyl}$ were derived from the plasmid pJZ, which was a gift from Nediljko Budisa and contains genes encoding a wild type $tRNA^{Pyl}$ and a N-terminally Strep-tagged mmPylRS evolved for the incorporation of BCN. The mmPylRS insert was cloned without Strep-tag with primers CH53 & AR365 and the $tRNA^{Pyl}$ insert with primers AR367 & AR368.

Phusion polymerase (Clontech) was used to generate the PCR fragments, that were assembled with the help of complementary primer overhangs in MegaPrimer PCRs and subsequently cloned into the backbone using InFusion Cloning (Takara Bio). The resulting vector pAC^{U}_BCN was used as template for multiple point mutations in the gene of *M. mazei* derived pyrrolysyl-tRNA synthetase to generate a variety of pAC^{U} vectors encoding evolved mmPylRS, specific for incorporation of various lysine-derivative ncAA.

The wild type $tRNA^{Pyl}$ contains an unfavourable U:G wobble pair in the anticodon stem, which was mutated to a C:G pair to improve suppression efficiency[11]. This mutagenesis was performed with primers CH57 & CH56.

The mbPylRS insert derived from the pAcBac1.tR4-MbPyl vector, which was a gift from Peter Schultz (Addgene plasmid # 50832) and contains genes encoding an undescribed mbPylRS and two copies of synthetic $tRNA^{Pyl}$ derived from *Desulfitobacterium hafniense*. The mbPylRS insert was cloned with primers CH51 & CH52. Same improved $tRNA^{Pyl}$ was used as for the *M. mazei* derived PylRS-$tRNA^{Pyl}$ pair.

Phusion polymerase (Clontech) was used to generate the PCR fragments, that were assembled with the help of complementary primer overhangs in MegaPrimer PCRs and subsequently cloned into the backbone using InFusion Cloning (Takara Bio) resulting in the pAC^{U}_PyLys vector. The *M. barkeri* derived pyrrolysyl-tRNA synthetase was only used in its wild type form.

2.2. Cloning of pAC^E vectors

Genes encoding different mutants of aaRSs in the pAC^U vectors were used as templates to create pAC^E plasmids (see Supplementary Fig. S1). The pAC^E backbone was derived from the pEVOL-pBpF vector and was amplified with primers CH123 & CH124. The insert between the two copies of the gene encoding aaRS was also derived from the pEVOL-pBpF vector and was cloned with primers CH100 & CH101.

2.2.1. *Methanococcus jannschii* derived TyrRS-$tRNA^{Tyr}$ pair

The two copies of the gene encoding mjTyrRS were derived from the corresponding pAC^U plasmids and were amplified with primers CH102 & CH103 and CH104 & CH105, and combined with the pEVOL insert in a MegaPrimer PCR using primers CH116 & CH118, and subsequently cloned into the backbone using InFusion Cloning (Takara Bio).

2.2.2. *Methanosarcina mazei/ barkeri* derived PylRS-$tRNA^{Pyl}$ pair

For the *M. mazei* and *M. barkeri* pAC^E plasmids, the gene encoding $tRNA^{Tyr}$ had to be replaced with the respective gene for $tRNA^{Pyl}$. Therefore, primers AR369 & AR366 were used to amplify the pAC^E backbone and primers AR367 & AR368 were for the $tRNA^{Pyl}$ insert, respectively. Again, the two copies of the gene encoding both aaRSs were derived from the corresponding pAC^U plasmids. For mmPylRS primers CH96 & CH97, and CH98 & CH99 were used to amplify a fragment, which was combined with the pEVOL insert in a MegaPrimer PCR using primers CH115 & CH117, and subsequently cloned into the backbone using InFusion Cloning (Takara Bio).

To amplify the gene encoding the mbPylRS primers CH106 & CH107 and CH108 & CH109 were used and the respective PCR product was combined with the pEVOL insert in a MegaPrimer PCR using primers CH115 & CH119, and subsequently cloned into the backbone using InFusion Cloning (Takara Bio).

Supplementary Data:

Mass spectrometric analysis of ACP-GFP constructs

Mass spectrometric analysis of purified ACP-GFP constructs was performed on a nanoESI (Synapt G2-S). For each construct the highest peak of the +12 ionized species was picked to calculate the protein mass. Within accuracy of the mass spectrometry device, found protein masses were typically 8-21 Da smaller than theoretical protein masses (except for ACP-GFP_Gln70AzPhe which showed a mass difference of 54 Da). The experimental mass differences between wild type and ACP-GFP with incorporated ncAAs were determined with a deviation of ±7 Da (except for ACP-GFP_Gln70AzPhe which showed a deviation of -39 Da). We do not have a definite explanation for the observed mass shift of the Gln70AzPhe mutant. Instability of the azido group during protein analysis may account for the reduced molecular weight, but cannot explain why this mass shift is only observed for this mutant. However, the Gln70AzPhe mutant can be specifically labelled in a SPAAC with the BCN-functionalized fluorophore, which confirms presence of the azido group at this stage (see Fig. 5 of the manuscript). Owing to this inconsistency, the Gln70 position was considered unreliable and not chosen in the full-length protein construct.

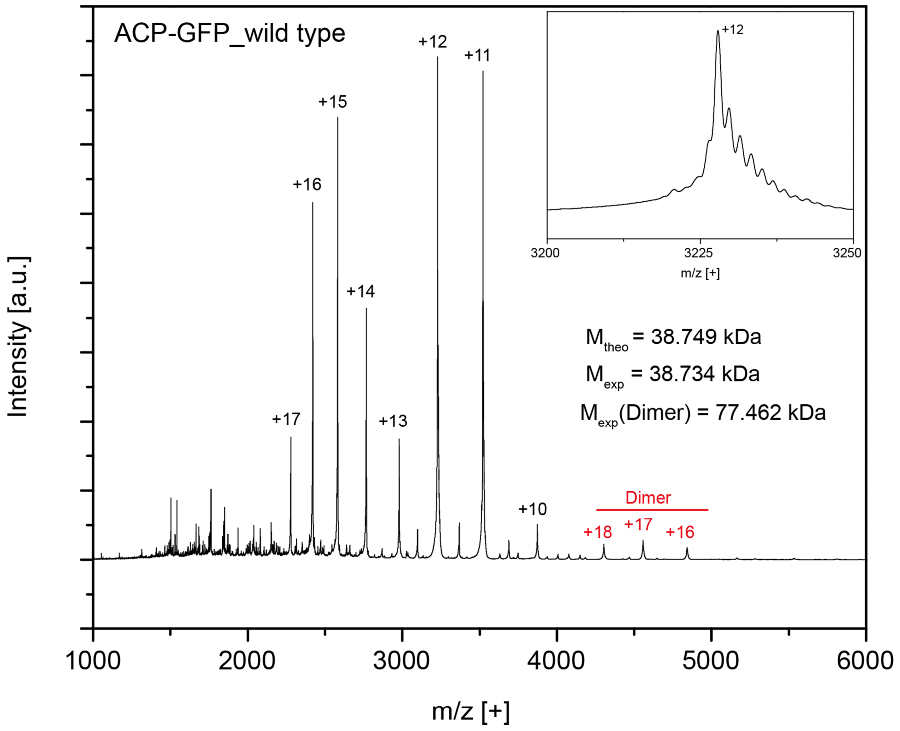
ACP-GFP_wild type
+12
+11
+15
+16
+14
+17
+13
+10
Dimer
+18
+17
+16
Intensity [a.u.]
m/z [+]
1000
2000
3000
4000
5000
6000
3200
3225
3250
M$_{theo}$ = 38.749 kDa
M$_{exp}$ = 38.734 kDa
M$_{exp}$(Dimer) = 77.462 kDa

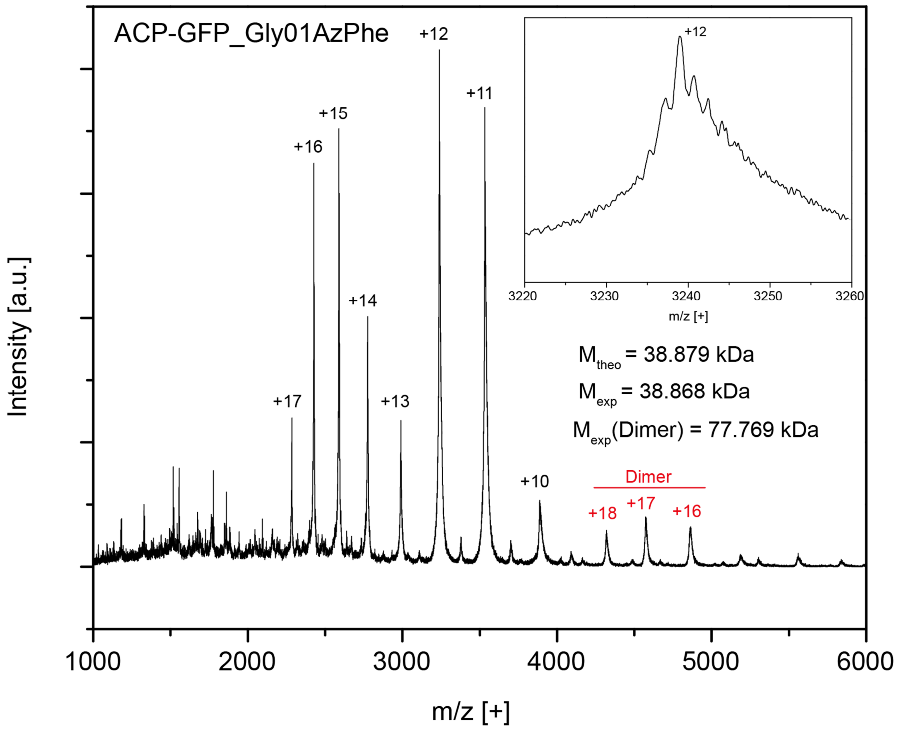
ACP-GFP_Gly01AzPhe
+12
+11
+15
+16
+14
+17
+13
+10
Dimer
+18
+17
+16
Intensity [a.u.]
m/z [+]
1000
2000
3000
4000
5000
6000
3220
3230
3240
3250
3260
M$_{theo}$ = 38.879 kDa
M$_{exp}$ = 38.868 kDa
M$_{exp}$(Dimer) = 77.769 kDa

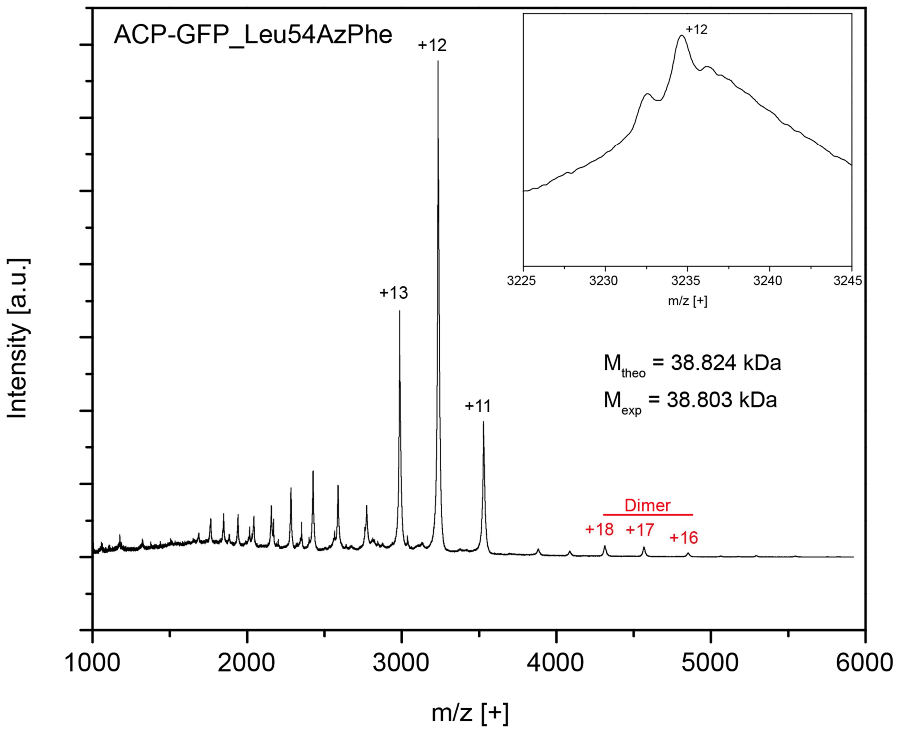
ACP-GFP_Leu54AzPhe
Intensity [a.u.]
m/z [+]
+12
+13
+11
Dimer
+18 +17 +16
M$_{theo}$ = 38.824 kDa
M$_{exp}$ = 38.803 kDa
1000 2000 3000 4000 5000 6000
3225 3230 3235 3240 3245

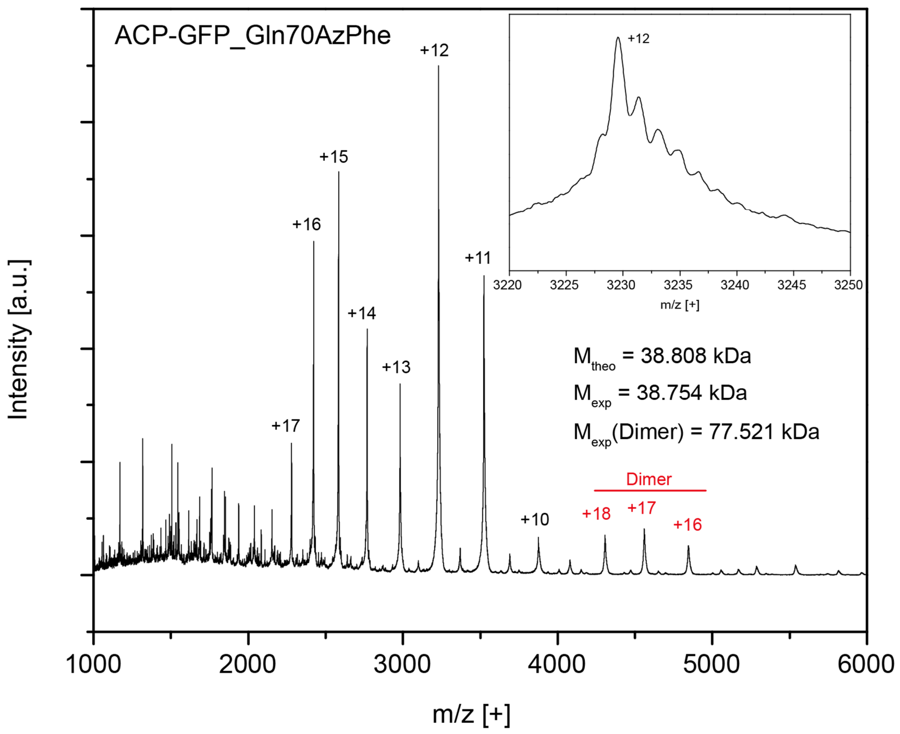
ACP-GFP_Gln70AzPhe
Intensity [a.u.]
m/z [+]
+12
+15
+16
+14
+13
+17
+11
+10
Dimer
+18 +17 +16
M$_{theo}$ = 38.808 kDa
M$_{exp}$ = 38.754 kDa
M$_{exp}$(Dimer) = 77.521 kDa
1000 2000 3000 4000 5000 6000
3220 3225 3230 3235 3240 3245 3250

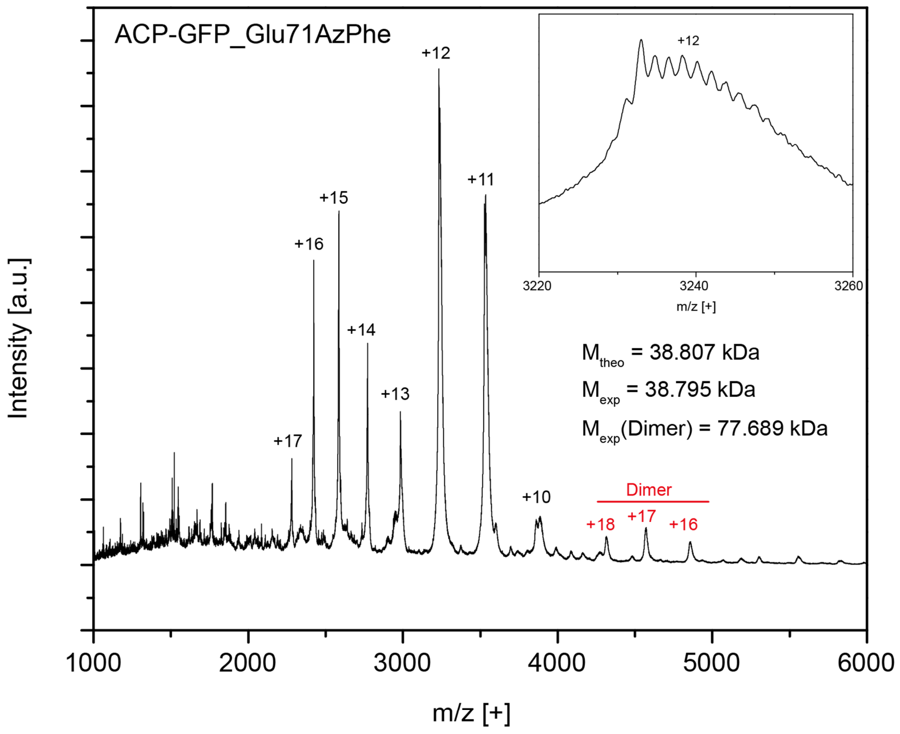
ACP-GFP_Glu71AzPhe
+12
+11
+15
+16
+14
+13
+17
+10
Dimer
+18
+17
+16
3220
3240
3260
m/z [+]
M_{theo} = 38.807 kDa
M_{exp} = 38.795 kDa
M_{exp}(Dimer) = 77.689 kDa
Intensity [a.u.]
1000
2000
3000
4000
5000
6000
m/z [+]

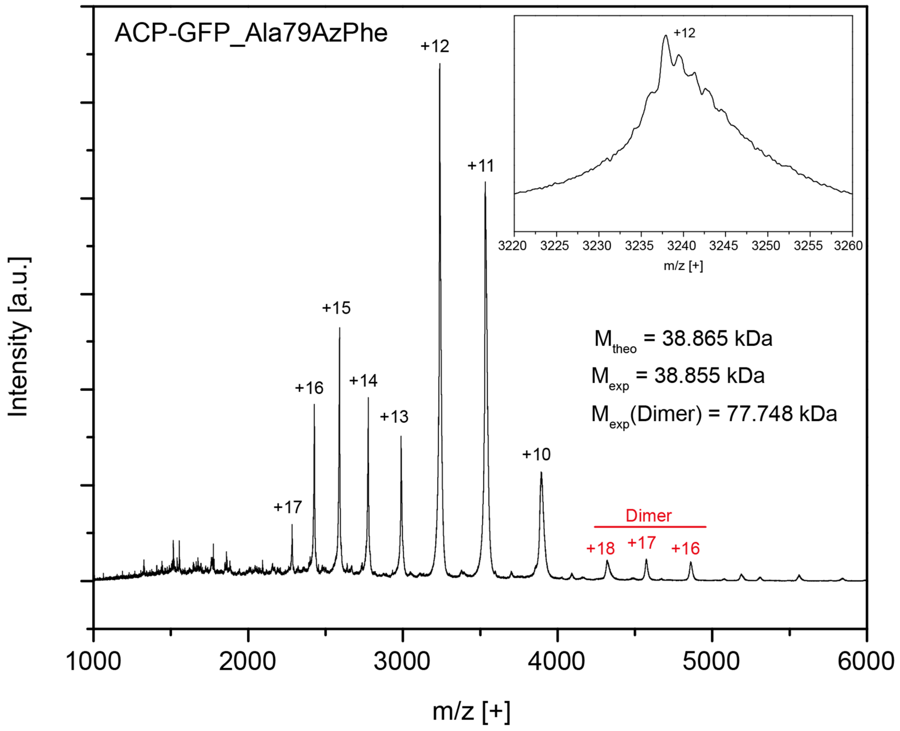
ACP-GFP_Ala79AzPhe
+12
+11
+15
+16
+14
+13
+17
+10
Dimer
+18
+17
+16
3220
3225
3230
3235
3240
3245
3250
3255
3260
m/z [+]
M_{theo} = 38.865 kDa
M_{exp} = 38.855 kDa
M_{exp}(Dimer) = 77.748 kDa
Intensity [a.u.]
1000
2000
3000
4000
5000
6000
m/z [+]

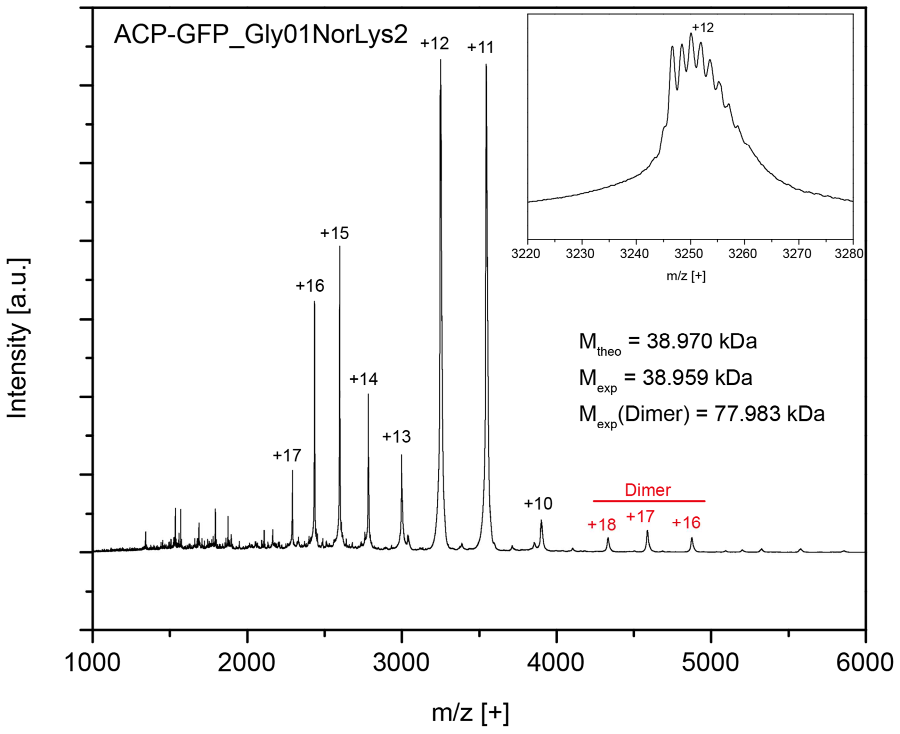
ACP-GFP_Gly01NorLys2
+12
+11
+15
+16
+14
+13
+17
+10
Dimer
+18
+17
+16
M_{theo} = 38.970 kDa
M_{exp} = 38.959 kDa
M_{exp}(Dimer) = 77.983 kDa
Intensity [a.u.]
m/z [+]
1000 2000 3000 4000 5000 6000
3220 3230 3240 3250 3260 3270 3280
m/z [+]

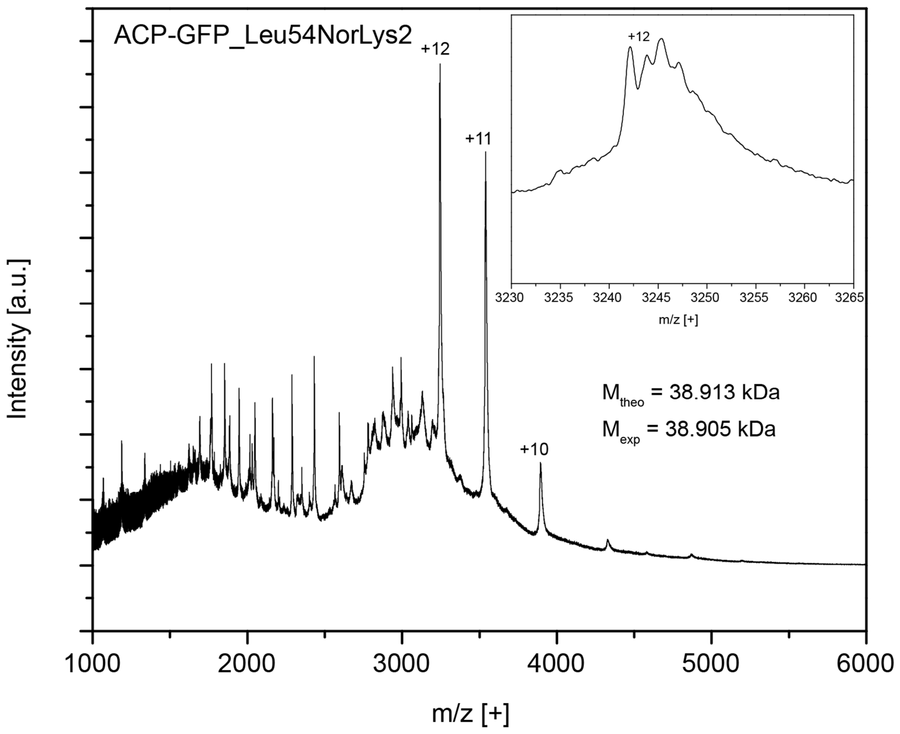
ACP-GFP_Leu54NorLys2
+12
+11
+10
M_{theo} = 38.913 kDa
M_{exp} = 38.905 kDa
Intensity [a.u.]
m/z [+]
1000 2000 3000 4000 5000 6000
3230 3235 3240 3245 3250 3255 3260 3265
m/z [+]

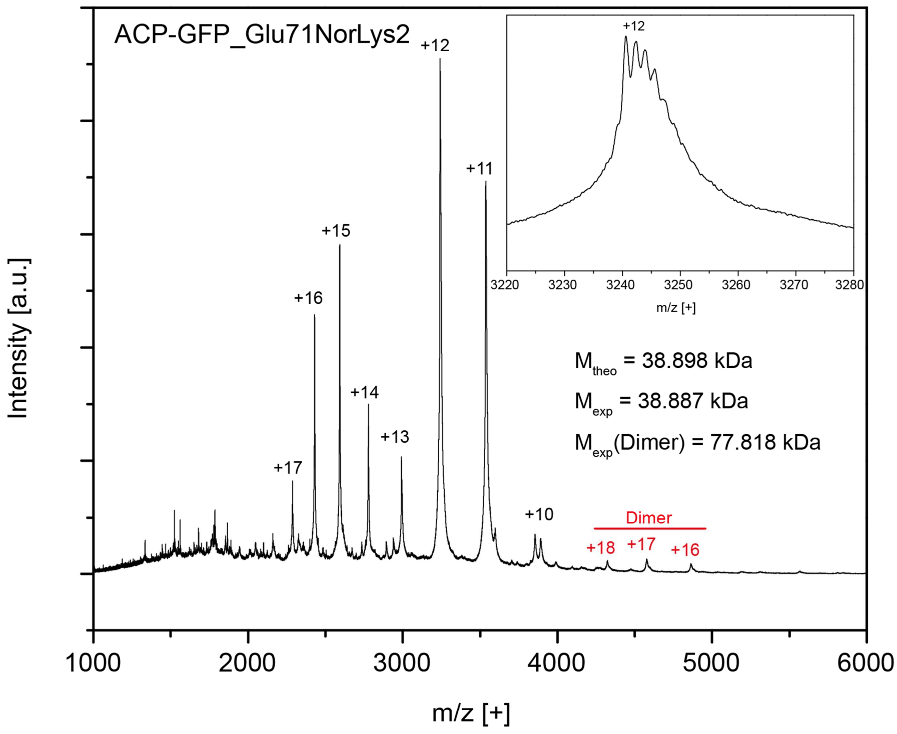

ACP-GFP_Glu71NorLys2
+12
+11
+15
+16
+14
+13
+17
+10
Dimer
+18 +17 +16
M_{theo} = 38.898 kDa
M_{exp} = 38.887 kDa
M_{exp}(Dimer) = 77.818 kDa
Intensity [a.u.]
m/z [+]
1000 2000 3000 4000 5000 6000
3220 3230 3240 3250 3260 3270 3280

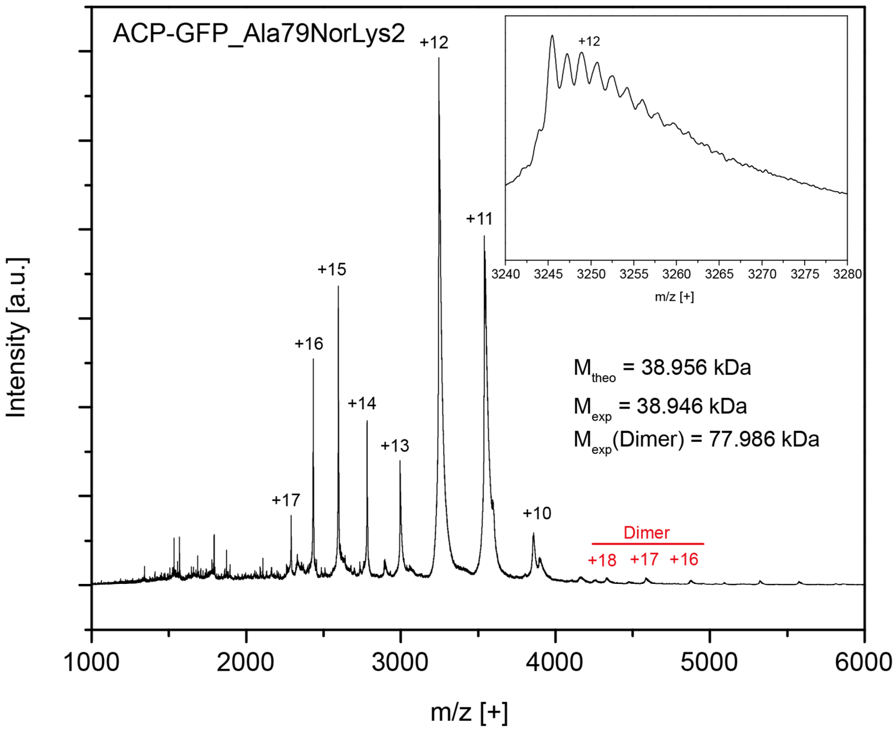

ACP-GFP_Ala79NorLys2
+12
+11
+15
+16
+14
+13
+17
+10
Dimer
+18 +17 +16
M_{theo} = 38.956 kDa
M_{exp} = 38.946 kDa
M_{exp}(Dimer) = 77.986 kDa
Intensity [a.u.]
m/z [+]
1000 2000 3000 4000 5000 6000
3240 3245 3250 3255 3260 3265 3270 3275 3280

Supplementary Figures

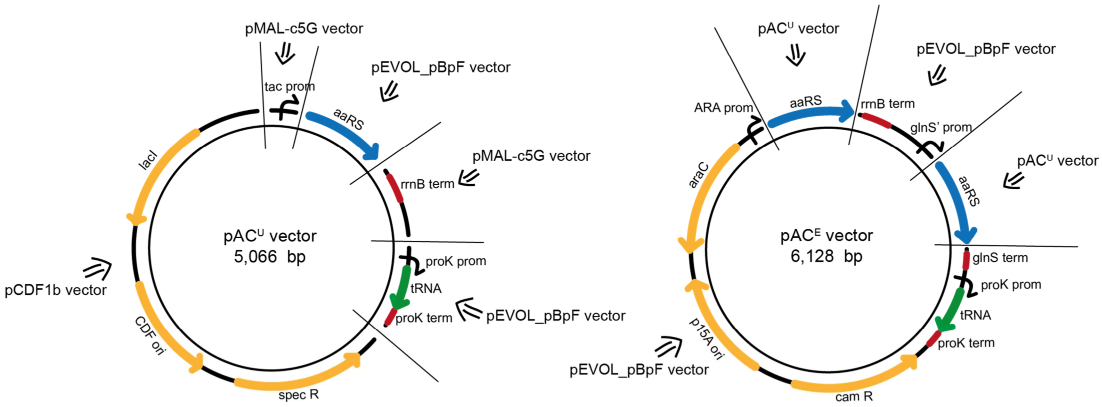

Figure S1: Cloning scheme of pAC^{U} and pAC^{E} vectors. The pAC^{U} vectors were constructed using the commercially available vectors pCDF1b, pMAL-c5G and pEVOL_pBpF (for mjTyrRS/tRNA^{Tyr} pair), pJZ (for mmPylRS/tRNA^{Pyl} pair) or pAcBac1.tR4-MbPyl (for mbPylRS/tRNA^{Pyl}) as templates. PCR fragments were assembled with help of complementary primer overhangs in MegaPrimer PCRs and subsequently cloned into the backbone using InFusion Cloning (Takara Bio). Multiple point mutations were introduced to create a set of genes encoding the different mutated aaRSs. The amber suppression library of pAC^{U} vectors was used as template to create pAC^{E} plasmids by replacing the genes encoding both copies of the aaRS in pEVOL_pBpF with the genes encoding respective mutants of aaRSs, using the same cloning methods.

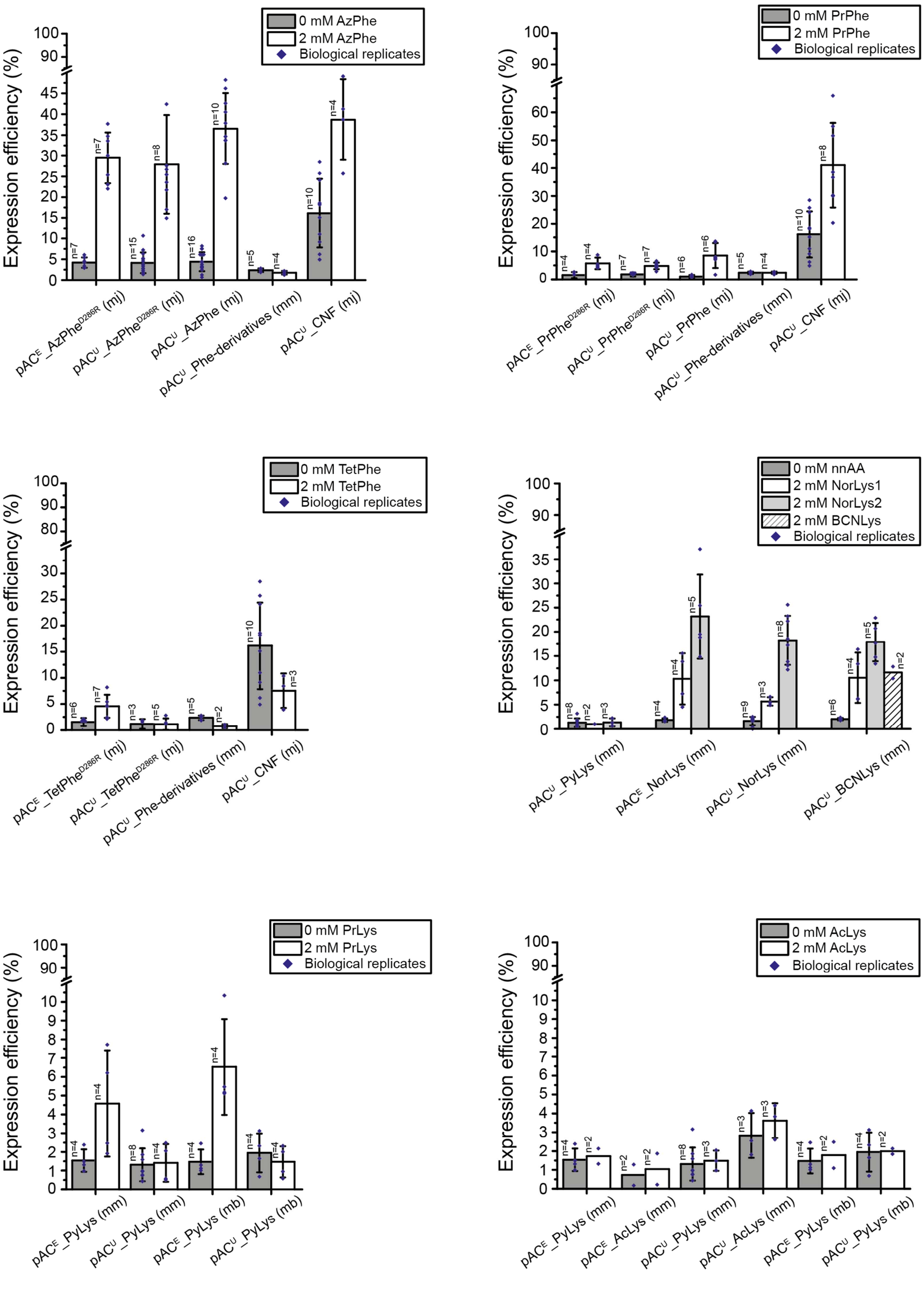

Figure S2: Screening for the most efficient suppression vector system to introduce select ncAAs with the reporter assay. The ACP-GFP construct with amber codon at site Leu54 was used for screening. Expression efficiency was read out by GFP fluorescence of 2 mL cell cultures and compared to the wild type reference. For incorporation, 2 mM ncAAs were supplemented to the medium. Cultures lacking ncAAs were taken as negative control to determine background signal. The averages of biological replicates are plotted together with standard deviation and the distribution of individual values is indicated as dots. Technical errors were below 10%.

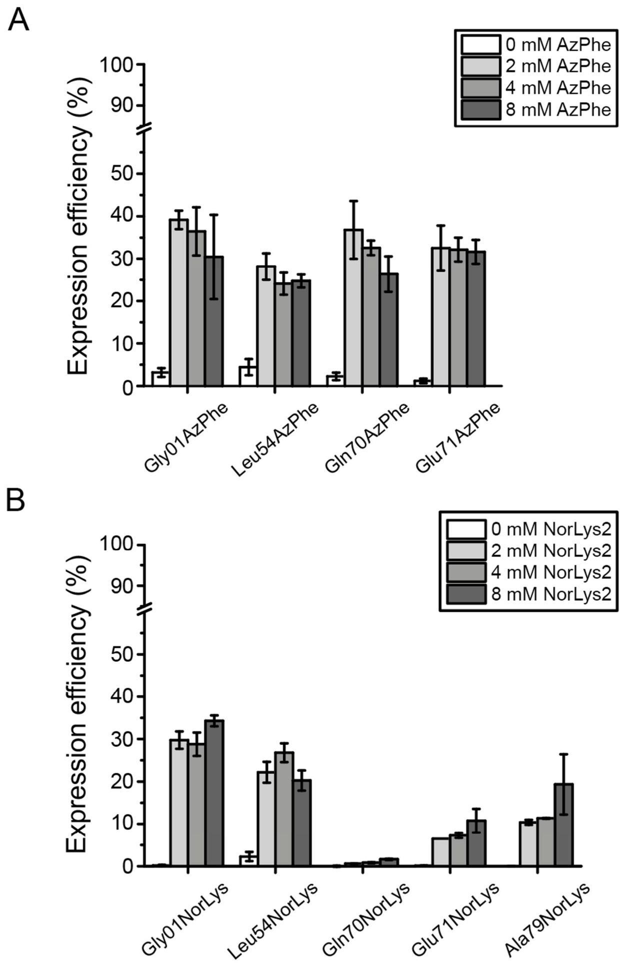

Figure S3: Testing the effect of different ncAA concentrations. Incorporation at different sites in the ACP-GFP construct was investigated with the reporter assay in 2 mL scale. Concentrations of 0, 2, 4 and 8 mM ncAA were supplemented to the medium. Expression efficiency was read out by GFP fluorescence and compared to the wild type reference. Error bars reflect the standard deviation of technical triplicates.

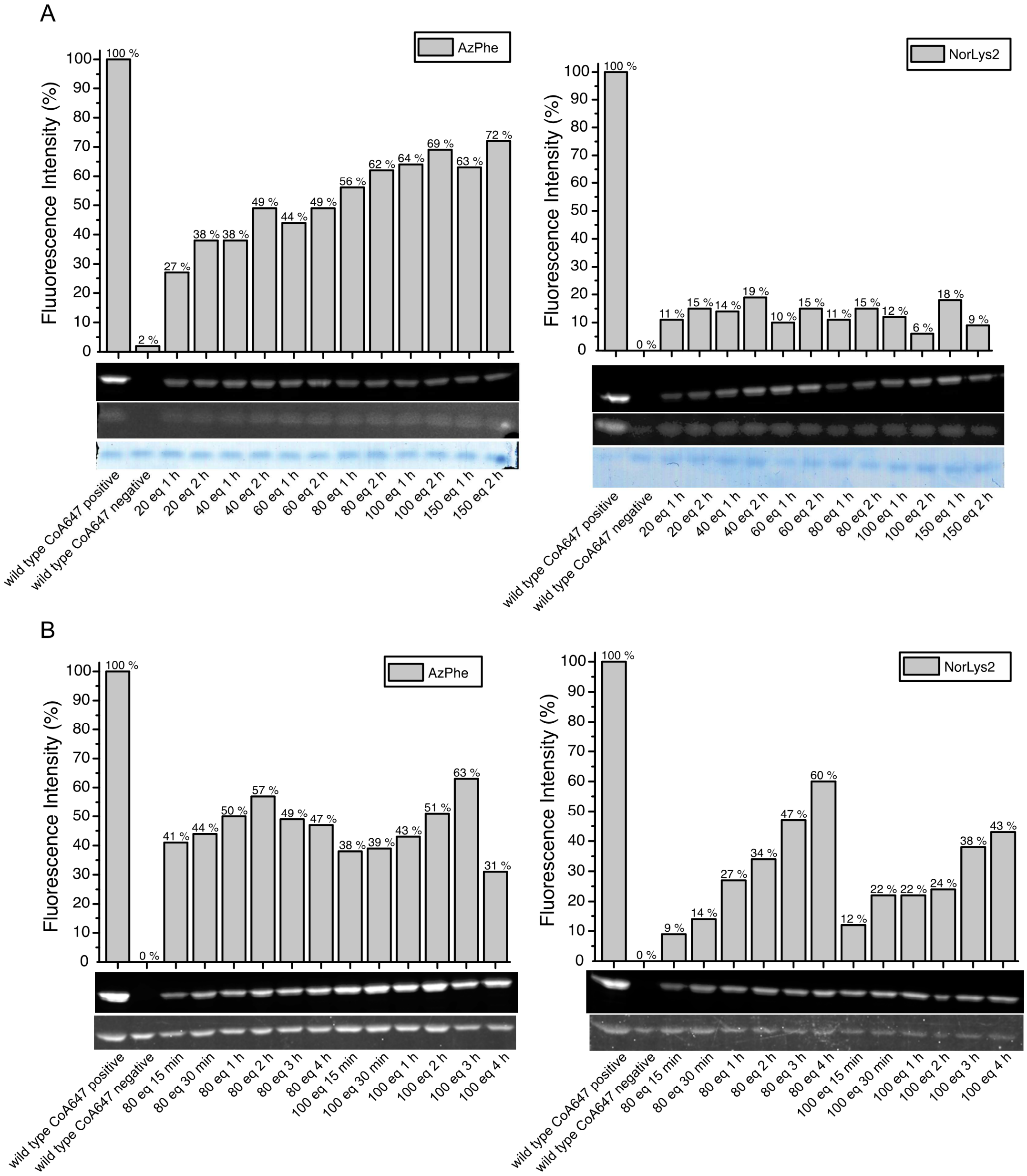

Figure S4: Optimizing the click reaction conditions for fluorescent labelling of ACP-GFP mutants. AzPhe mutants were labelled with BCN-POE$_3$-NH-DY649P1, NorLys2 mutants were labelled with 6-methyl-tetrazine-ATTO-647N and the wild type ACP-GFP was modified enzymatically with a fluorescent CoA647-label by Sfp. DOL determined by relative in-gel fluorescence intensities at wavelength 650 nm compared to wild type ACP-GFP reference. All fluorescence intensities were corrected by the quantum efficiencies of the respective fluorophores and correlated to the protein bands of the Syprored- or Coomassie-stained gel. A) DOL of AzPhe mutants (left panel) and NorLys2 mutants (right panel) monitored in dependence of different fluorophore equiv. after labelling reactions for 1 h and 2 h. B) Time dependence of the DOL of AzPhe mutants (left panel) and NorLys2 mutants (right panel) monitored for labelling reactions with 80 and 100 equiv. of fluorophore.

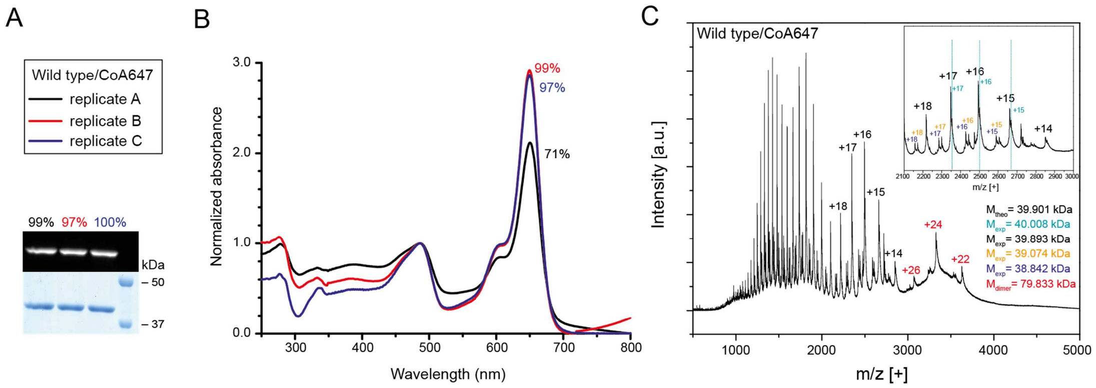

Figure S5: Quantification of *in vitro* phosphopantetheinylation of wild type ACP-GFP with CoA 647-label by Sfp. A) In-gel fluorescence intensities of wild type ACP-GFP CoA 647 conjugates. Wild type ACP-GFP was enzymatically modified with a fluorescent CoA 647-label by Sfp in three parallel reactions. In-gel fluorescence intensities were detected at wavelength 650 nm, corrected by the quantum efficiency of the DY647P1 fluorophore and correlated to the protein bands of the Coomassie-stained gel. Scans of the original gels are presented in Supplementary Fig. S6. B) DOL of wild type ACP-GFP CoA 647 conjugates determined by UV-Vis spectroscopy. Free fluorophore was removed by purification over Ni-NTA magnetic beads. UV-Vis absorbance spectra were normalized to GFP absorbance at 485 nm wavelength. DOL is read out by comparing absorbance of the fluorophore at 650 nm to absorbance of GFP at 485 nm. C) MS analysis of wild type ACP-GFP CoA 647 conjugates. Free fluorophore was removed by purification over spin column (Amicon Ultra 30K centrifugal filter). No unmodified wild type ACP-GFP mass was detected within the detection range of the instrument. The main peaks match the theoretical mass of modified wild type ACP-GFP CoA 647 conjugate. Impurities in MS spectra sum up to approximately 10%, which cannot be assigned to specific compound.

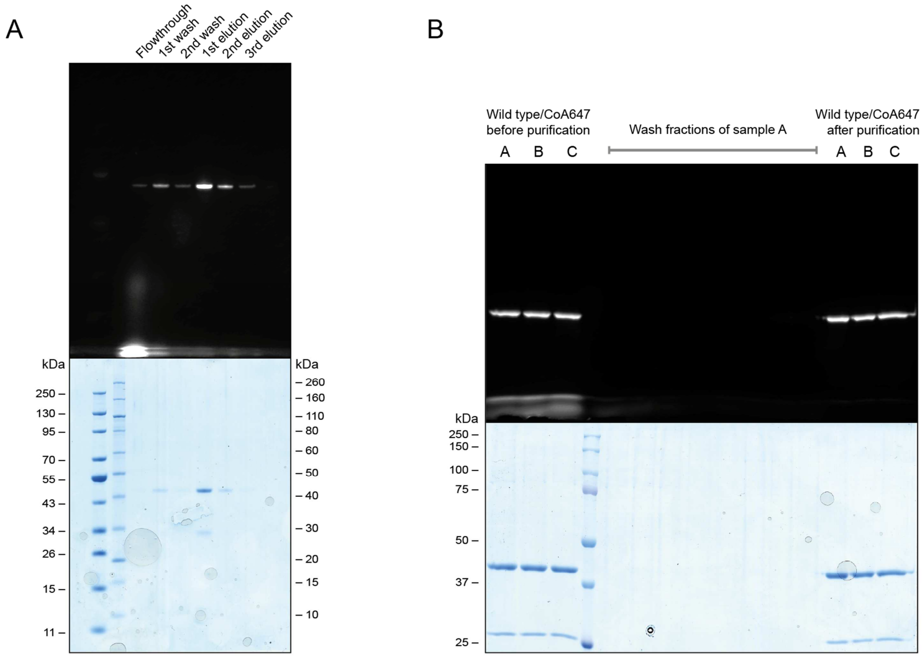

Figure S6: Removal of excess free fluorophore. A) SDS-PAGE (NuPAGE Bis-Tris 4-12%) gel of wild type ACP-GFP CoA 647 conjugate purified over Ni-NTA magnetic beads. Non-covalently bound dye molecules are washed off. The elution fractions show only protein dye conjugate and no free fluorophore. B) SDS-PAGE (polyacrylamide 12%) gel of wild type ACP-GFP CoA 647 conjugate purified over spin column. Wild type ACP-GFP was enzymatically modified with a fluorescent CoA647-label by Sfp (additional band at 26 kDa) in three parallel reactions. For MS analysis, the protein buffer was exchanged by 0.1 M ammonium acetate buffer in an Amicon Ultra 30K centrifugal filter device. After eight centrifugation steps, non-covalently bound dye molecules are washed off. In-gel fluorescence intensities were detected at wavelength 650 nm.

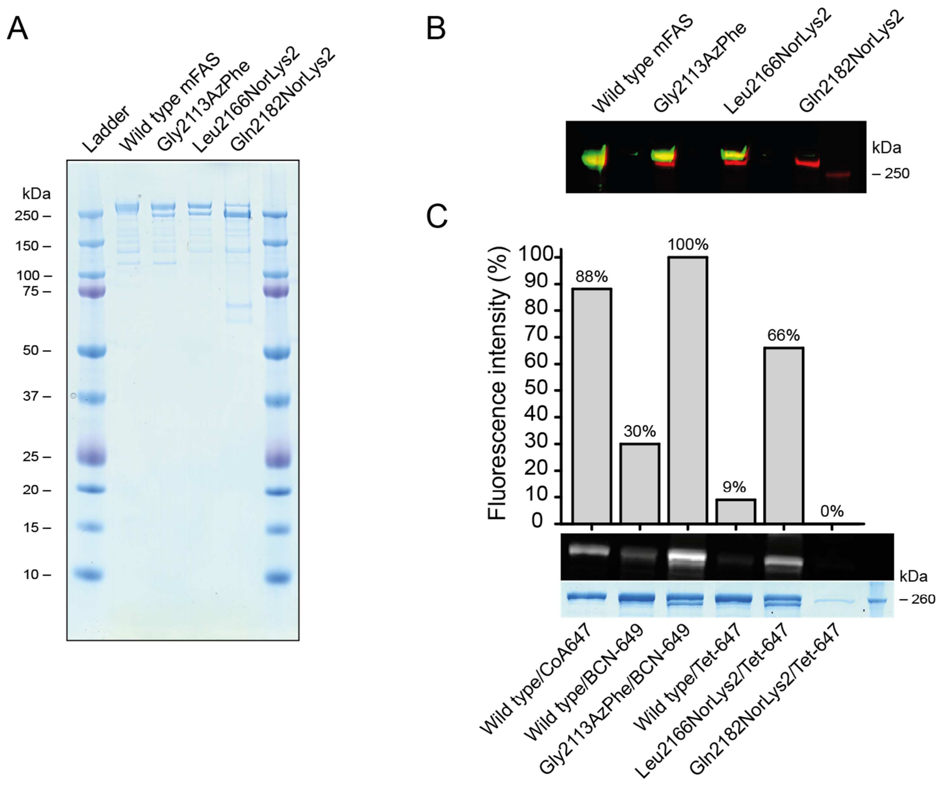

Figure S7: Production of ncAA-modified mFAS mutants. A) SDS-PAGE (NuPAGE Bis-Tris 4-12%) gel of mFAS mutants. Proteins were purified by a tandem affinity chromatography via the N-terminal Strep-tag and the C-terminal His-tag. B) Quantitative western blot of mFAS mutants. The red channel refers to antibodies conjugated with DyLight 755 against the N-terminal Strep-tag and the green channel to antibodies conjugated with DyLight 633 against the C-terminal His-tag, respectively. The missing green band in lane 4 indicates that expression of full-length Gln2182NorLys2 mFAS failed and that only a truncated construct without the C-terminal part was obtained. C) Fluorescent labelling of mFAS mutants. AzPhe mutants were labelled with BCN-POE$_3$-NH-DY649P1 (BCN-649), NorLys2 mutants were labelled with 6-methyl-tetrazine-ATTO-647N (Tet-647) and the wild type mFAS was enzymatically modified at the ACP domain by a fluorescent CoA647-label by Sfp. DOL is determined by the relative in-gel fluorescence intensities at wavelength 650 nm related to the wild type reference. All fluorescence intensities were corrected by quantum efficiency of the respective fluorophore and correlated to the protein bands of the Coomassie-stained gel.

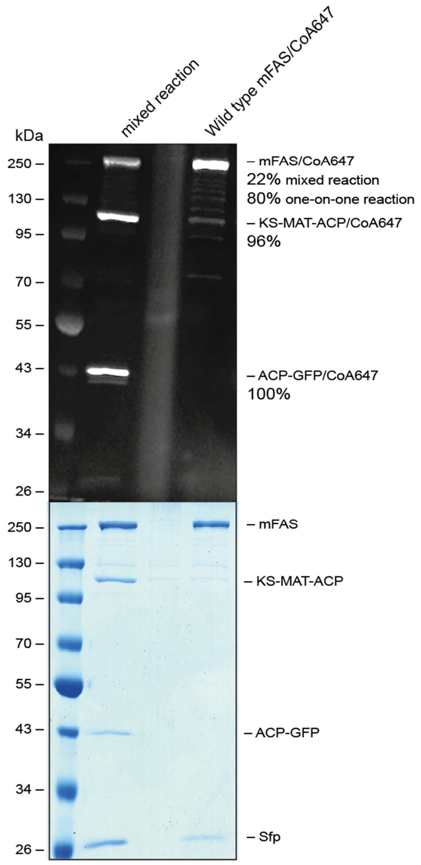

Figure S8: *In vitro* phosphopantetheinylation of wild type mFAS with CoA 647-label by Sfp. In-gel fluorescence intensities of different *in vitro* phosphopantetheinylation reactions with CoA 647-label were compared. In a one-on-one reaction, 1 equiv. wild type mFAS was treated with 1 equiv. Sfp and 5 equiv. CoA 647 fluorohpore. In a mixed reaction, equimolar amounts (1.85 µM each, 5.55 µM total protein) of wild type mFAS, wild type ACP-GFP and of a KS-MAT protein construct with fused ACP domain, were treated with 1 equiv. Sfp and 5 equiv. CoA 647 fluorophore. *In vitro* phosphopantetheinylation of wild type mFAS seems to be less efficient than the reaction of ACP-GFP or KS-MAT-ACP. In-gel fluorescence intensities were detected at wavelength 650 nm, corrected by the quantum efficiency of the DY647P1 fluorophore and correlated to the protein bands of the Coomassie-stained gel.

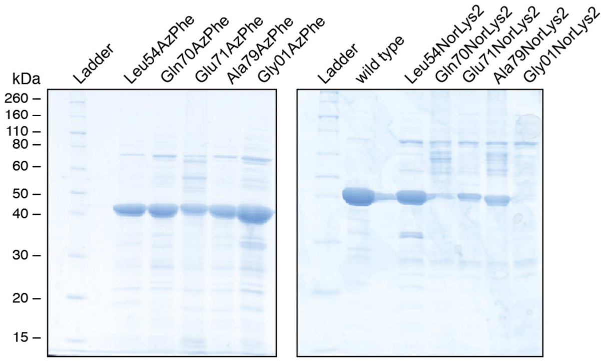

Figure S9: Large scale expression and purification of ACP-GFP mutants. Original SDS-PAGE (NuPAGE Bis-Tris 4-12%) gel of ACP-GFP mutants purified by Ni-chelating chromatography. Referring to Fig. 4 in the manuscript.

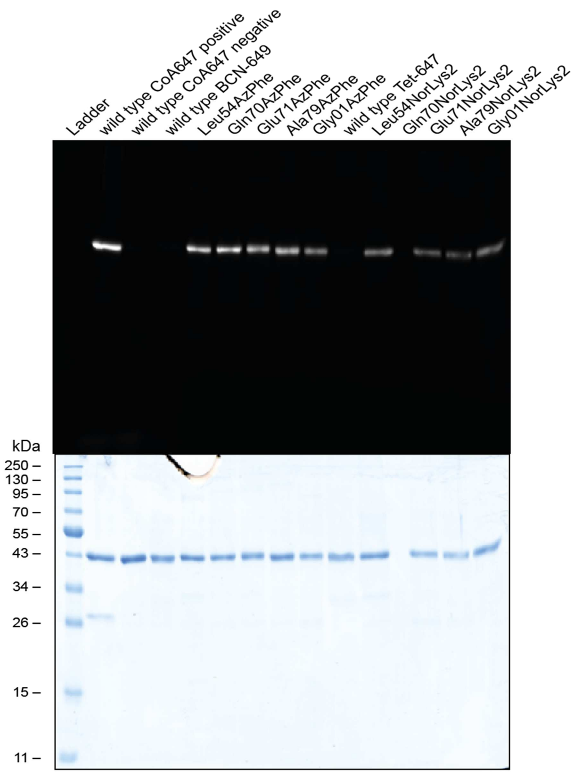

Figure S10: Fluorescent labelling of ACP-GFP mutants. Original fluorescent gel and Coomassie-stained gel (polyacrylamide 12%) of fluorescently labelled ACP-GFP mutants. Referring to Fig. 5 in the manuscript.

Supplementary Tables

Table S1: List of primers used for cloning amber codon suppression vectors pAC^U and pAC^E.

#	Primer name	Primer sequence
CH02	pCDF1b_for	CTGAAACCTCAGGCATTTGAGAAG
CH03	pCDF1b_rev	ATTTCCTAATGCAGGAGTCGCATAAG
CH04	pCDF_tac_for	CCTGCATTAGGAAATTTGACAATTAATCATCGGCTCGTATAATG
CH05	tac_Nde1-rev	ATGCTATGGTCCTTGTTGGTCAATTGC
CH06	tac_mjTyrS_for	CAATTGACCAACAAGGACCATAGCATATGGACGAATTTGAAATGATAAAGAGAAAC
CH11	mjtRNA_pCDF1b_rev	TGCCTGAGGTTTCAGCAAATTCGACCCTGAGCTGC
CH19	mjtRNA_rrnBterm_rev	GCGGAATTATTTGATCGGCGAGGTCTCATGAGCGGATACATATTTG
CH32	rrnBterm_for	GGTGCGGCACACTACGTAC
CH33	mjTyrS_rrnBterm_rev	GTACGTAGTGTGCCGCACCTTATAATCTCTTTCTAATTGGCTCTAAAATC
CH34	rrnBterm_mjtRNA_for	CTCGCCGATCAAATAATTCCGCTAATTCCGCTTCGCAACATGTGAG
CH51	tac_mbPylRS	CAAGGACCATAGCATATGGATAAAAAACCATTAGATGTTTTAATATCTGC
CH52	mbPylRS_rrnBterm_rev	GTAGTGTGCCGCACCTCATAGATTGGTTGAAATCCCATTATAGTAAG
CH53	tac_notag-mmPylRS_for	CAAGGACCATAGCATATGGATAAAAAACCACTAAACACTCTGATATC
CH56	PyltRNA_wobble_rev	TTCGATCTACATGATCAGGTTTCCAATG
CH57	PyltRNA_wobble_for	ATCATGTAGATCGAAcGGACTCTAAATCCGTTCAGCC
CH96	ara_mmPylRS_forward	GGAGGAATTAGATCTATGGATAAAAAACCACTAAACACTCTGATATCTG
CH97	mmPylRS_rrnB_rev	CTCAATGATGATGATGATGATGGTCGACTTATTACAGGTTGGTAGAAATCCCGTTATAG
CH98	glnsprom_mmPylRS_for	CGTTGTTTACGCTTTGAGGAATCCCATATGGATAAAAAACCACTAAACACTCTGATATCTG
CH99	mmPylRS_glnsterm_reverse	CGTTTGAAACTGCAGTTACAGGTTGGTAGAAATCCCGTTATAG
CH100	pEVOL_insert_forward	TAAGTCGACCATCATCATCATCATC
CH101	pEVOL_insert_reverse	ATGGGATTCCTCAAAGCGTAAACAAC
CH102	ara_mjTyrRS_forward	GGAGGAATTAGATCTATGGACGAATTTGAAATGATAAAGAGAAAC
CH103	mjTyrRS_rrnB_rev	CTCAATGATGATGATGATGATGGTCGACttaTTATAATCTCTTTCTAATTGGCTCTAAAATCTTTA
CH104	glnsprom_mjTyrRS_for	CGTTGTTTACGCTTTGAGGAATCCCATATGGACGAATTTGAAATGATAAAGAGAAAC
CH105	mjTyrRS_glnsterm_rev	CGTTTGAAACTGCAGTTATAATCTCTTTCTAATTGGCTCTAAAATCTTTA
CH106	ara_mbPylRS_for	GGAGGAATTAGATCTATGGATAAAAAACCATTAGATGTTTTAATATCTG
CH107	mbPylRS_rrnBterm_rev	CTCAATGATGATGATGATGATGGTCGACTTATCATAGATTGG

		TTGAAATCCCATTATAG
CH108	glnsprom_mbPylRS_for	CGTTGTTTACGCTTTGAGGAATCCCATATGGATAAAAAACCATTAGATGTTTTAATATCTG
CH109	mbPylRS_glnsterm_rev	CGTTTGAAACTGCAGTCATAGATTGGTTGAAATCCCATTATAG
CH115	MegaPrimer_mmpEvol_for	GGAGGAATTAGATCTATGGATAAAAAAC
CH116	MegaPrimer_mjpEvol_for	GGAGGAATTAGATCTATGGACGAATTTG
CH117	MegaPrimer_mmpEvol_rev	CGTTTGAAACTGCAGTTACAGGTTG
CH118	MegaPrimer_mjpEvol_rev	CGTTTGAAACTGCAGTTATAATCTCTTTC
CH119	MegaPrimer_mbpEvol_rev	CGTTTGAAACTGCAGTCATAGATTGG
CH123	pEvol_backbone_for	CTGCAGTTTCAAACGCTAAATTGCC
CH124	pEvol_backbone_rev	AGATCTAATTCCTCCTGTTAGCCC
AR365	mmPylS_rrnB_rev	GTACGTAGTGTGCCGCACCTTACAGGTTGGTAGAAATCCCGTTATAG
AR366	proK_rev	AATGCGGGGCGCATCTTACTG
AR367	proK_mmtRNA_for	CAGTAAGATGCGCCCCGCATTGGAAACCTGATCATGTAGATCGAATG
AR368	mmtRNA_terK_rev	GCTTTTCGAATTTGGTGGCGGAAACCCCGGGAATC
AR369	terK_for	CCAAATTCGAAAAGCCTGCTCAAC

Table S2: List of amber codon suppressor plasmids used in this study.

#	Plasmid name	aaRS variant	ncAA
1	pAC^{U}_AzPhe (mj)	*Methanococcus jannaschii* tyrosyl-tRNA synthetase[12] Y32T, E107N, D158P, I159L, L162Q	AzPhe
2	pAC^{U}_AzPhe^{D286R} (mj)	*Methanococcus jannaschii* tyrosyl-tRNA synthetase[12,13] Y32T, E107N, D158P, I159L, L162Q, D286R	
3	pAC^{E}_AzPhe^{D286R} (mj)		
4	pAC^{U}_PrPhe (mj)	*Methanococcus jannaschii* tyrosyl-tRNA synthetase[14] Y32A, E107P, L110F, D158A, L162A	PrPhe
5	pAC^{U}_PrPhe^{D286R} (mj)	*Methanococcus jannaschii* tyrosyl-tRNA synthetase[13,14] Y32A, E107P, L110F, D158A, L162A, D286R	
6	pAC^{E}_PrPhe^{D286R} (mj)		
7	pAC^{U}_TetPhe^{D286R} (mj)	*Methanococcus jannaschii* tyrosyl-tRNA synthetase[5,13] Y32E, L65A, F108P, Q109S, D158G, L162G, D286R	TetPhe
8	pAC^{E}_TetPhe^{D286R} (mj)		
9	pAC^{U}_CNF (mj)	*Methanococcus jannaschii* tyrosyl-tRNA synthetase[13,15] Y32L, F108W, Q109M, D158G, I159A, D286R	AzPhe; PrPhe; TetPhe
10	pAC^{U}_PyLys (mm)	*Methanosarcina mazei* pyrrolysyl-tRNA synthetase[16]	NorLys1; NorLys2; PrLys; AcLys
11	pAC^{E}_PyLys (mm)		
12	pAC^{U}_AcLys (mm)	*Methanosarcina mazei* pyrrolysyl-tRNA synthetase[17] L301M, Y306L, L309A, C348F	AcLys
13	pAC^{E}_AcLys (mm)		
14	pAC^{U}_NorLys (mm)	*Methanosarcina mazei* pyrrolysyl-tRNA synthetase[18] Y306G, Y384F, I405R	NorLys1; NorLys2
15	pAC^{E}_NorLys (mm)		
16	pAC^{U}_BCN (mm)	*Methanosarcina mazei* pyrrolysyl-tRNA synthetase[19] Y306A, Y348A	BCNLys; NorLys1; NorLys2
17	pAC^{U}_Phe-derivatives (mm)	*Methanosarcina mazei* pyrrolysyl-tRNA synthetase[20] N346A, C348A	AzPhe; PrPhe; TetPhe
18	pAC^{U}_PyLys (mb)	*Methanosarcina barkeri* pyrrolysyl-tRNA synthetase[21]	PrLys; AcLys
19	pAC^{E}_PyLys (mb)		

Table S3: List of expression plasmids for ACP-GFP constructs used in this study. All genes of ACP-GFP constructs are encoded on pET22b vectors with ampicillin resistance and stand under a T7 promoter. Monomeric superfolder GFP was fused C-terminally to the ACP domain. All ACP-GFP fusion constructs contain a N-terminal StrepII-tag and C-terminal His8-tag. The Met2184Leu mutation prevents an alternative translation start and reduces background GFP-fluorescence in the reporter assay. Mutation sites are numbered according to the mFAS sequence (UniProtKB accession code P19096).

ACP-GFP constructs		
**#**	**Plasmid name**	**Mutation**
pCH92	T7_StrepII_mACP(M2184L)_msfGFP_H8_pET22b	Met2183Leu
pCH91	T7_StrepII_(ALAmut)_mACP(M2184L)_msfGFP-H8_pET22b	Met2184Leu<br>Amber mutation at site Ala-1
pBG05	T7_StrepII_mACP(G2113mut_M2184L)_msfGFP-H8_pET22b	Met2184Leu<br>Amber mutation at site Gly2113
pBG01	T7_StrepII_mACP(L2166mut_M2184L)_msfGFP-H8_pET22b	Met2184Leu<br>Amber mutation at site Leu2166
pBG02	T7_StrepII_mACP(Q2182mut_M2184L)_msfGFP-H8_pET22b	Met2184Leu<br>Amber mutation at site Gln2182
pBG03	T7_StrepII_mACP(E2183mut_M2184L)_msfGFP-H8_pET22b	Met2184Leu<br>Amber mutation at site Glu2183
pBG04	T7_StrepII_mACP(M2184L_A2191mut)_msfGFP-H8_pET22b	Met2184Leu<br>Amber mutation at site Ala2191

Table S4: List of expression plasmids for mFAS constructs used in this study. All genes of mFAS constructs are encoded on pET22b vectors with ampicillin resistance and stand under a T7 promoter. All mFAS constructs contain a N-terminal StrepI-tag and C-terminal His8-tag.

mFAS constructs		
**#**	**Plasmid name**	**Mutation**
pAR18	T7_StrepI_mFASm_H8_pET22b	Wild type
pBG09	T7_StrepI_mFASm(G2113mut)_H8_pET22b	Amber mutation at site Gly2113
pBG10	T7_StrepI_mFASm(L2166mut)_H8_pET22b	Amber mutation at site Leu2166
pBG06	T7_StrepI_mFASm(Q2182mut)_H8_pET22b	Amber mutation at site Gln2182
pAR378	T7_StrepI_Not1_mFASm(S2150A_G2113X)_H8_pET22b	Amber mutation at site Gly2113<br>and ACP knock out

References

1 Wieland, T., von Dungen, A. & Birr, C. Synthese einer antitoxischen Antamid-Variante mit *p*-Azido-phenylalanin in Stelle 6. *Liebigs Ann. Chem.* **752**, 109-114 (1971).

2 Lieber, E., Levering, D. R. & Patterson, L. Infrared Absorption Spectra of Compounds of High Nitrogen Content. *Anal. Chem.* **23**, 1594-1604 (1951).

3 Jain, S. & Reiser, O. Immobilization of Cobalt(II) Schiff Base Complexes on Polystyrene Resin and a Study of Their Catalytic Activity for the Aerobic Oxidation of Alcohols. *ChemSusChem* **1**, 534-541, doi:10.1002/cssc.200800025 (2008).

4 Borzilleri, R., Zhang, Y., Miller, M. M. & Seigal, B. A. Preparation of macrocyclic compounds for inhibition of inhibitors of apoptosis. (2014).

5 Seitchik, J. L. *et al.* Genetically Encoded Tetrazine Amino Acid Directs Rapid Site-Specific *in Vivo* Bioorthogonal Ligation with *trans*-Cyclooctenes. *J. Am. Chem. Soc.* **134**, 2898-2901, doi:10.1021/ja2109745 (2012).

6 Fields, S. C., Parker, M. H. & Erickson, W. R. A simple route to unsymmetrically substituted 1, 2, 4, 5-tetrazines. *J. Org. Chem.* **59**, 8284-8287 (1994).

7 Nguyen, D. P. *et al.* Genetic Encoding and Labeling of Aliphatic Azides and Alkynes in Recombinant Proteins *via* a Pyrrolysyl-tRNA Synthetase/$tRNA_{CUA}$ Pair and Click Chemistry. *J. Am. Chem. Soc.* **131**, 8720-8721 (2009).

8 Khan, F. A. & Choudhury, S. An Efficient Synthesis of Substituted *meta*-Halophenols and Their Methyl Ethers: Insight into the Reaction Mechanism. *Eur. J. Org. Chem.* **2010**, 2954-2970 (2010).

9 Lang, K. *et al.* Genetically encoded norbornene directs site-specific cellular protein labelling via a rapid bioorthogonal reaction. *Nat. Chem.* **4**, 298-304, doi:10.1038/nchem.1250 (2012).

10 Kaya, E. *et al.* A Genetically Encoded Norbornene Amino Acid for the Mild and Selective Modification of Proteins in a Copper-Free Click Reaction. *Angew. Chem. Int. Ed.* **51**, 4466-4469, doi:10.1002/anie.201109252 (2012).

11 Chatterjee, A., Sun, S. B., Furman, J. L., Xiao, H. & Schultz, P. G. A versatile platform for single- and multiple-unnatural amino acid mutagenesis in *Escherichia coli*. *Biochemistry* **52**, 1828-1837, doi:10.1021/bi4000244 (2013).

12 Chin, J. W. *et al.* Addition of *p*-Azido-L-phenylalanine to the Genetic Code of *Escherichia coli*. *J. Am. Chem. Soc.* **124**, 9026-9027, doi:10.1021/ja027007w (2002).

13 Kobayashi, T. *et al.* Structural basis for orthogonal tRNA specificities of tyrosyl-tRNA synthetases for genetic code expansion. *Nat. Struct. Biol.* **10**, 425-432 (2003).

14 Deiters, A. & Schultz, P. G. *In vivo* incorporation of an alkyne into proteins in *Escherichia coli*. *Bioorg. Med. Chem. Lett.* **15**, 1521-1524, doi:10.1016/j.bmcl.2004.12.065 (2005).

15 Young, D. D. *et al.* An Evolved Aminoacyl-tRNA Synthetase with Atypical Polysubstrate Specificity. *Biochemistry* **50**, 1894-1900, doi:10.1021/bi101929e (2011).

16 Mukai, T. *et al.* Adding L-lysine derivatives to the genetic code of mammalian cells with engineered pyrrolysyl-tRNA synthetases. *Biochem. Biophys. Res. Commun.* **371**, 818-822 (2008).

17 Umehara, T. *et al.* *N*-Acetyl lysyl-tRNA synthetases evolved by a CcdB-based selection possess *N*-acetyl lysine specificity *in vitro* and *in vivo*. *FEBS Lett.* **586**, 729-733, doi:10.1016/j.febslet.2012.01.029 (2012).

18 Schneider, S. *et al.* Structural Insights into Incorporation of Norbornene Amino Acids for Click Modification of Proteins. *ChemBioChem* **14**, 2114-2118, doi:10.1002/cbic.201300435 (2013).

19 Plass, T. *et al.* Amino Acids for Diels-Alder Reactions in Living Cells. *Angew. Chem. Int. Ed.* **51**, 4166-4170, doi:10.1002/anie.201108231 (2012).

20 Wang, Y.-S., Fang, X., Wallace, A. L., Wu, B. & Liu, W. R. A Rationally Designed Pyrrolysyl-tRNA Synthetase Mutant with a Broad Substrate Spectrum. *J. Am. Chem. Soc.* **134**, 2950-2953, doi:10.1021/ja211972x (2012).

21 Blight, S. K. *et al.* Direct charging of $tRNA_{CUA}$ with pyrrolysine *in vitro* and *in vivo*. *Nature* **431**, 333-335 (2004).
